# Phytochemistry and Biological Activities of Essential Oils from Six Aromatic Medicinal Plants with Cosmetic Properties

**DOI:** 10.3390/antibiotics12040721

**Published:** 2023-04-07

**Authors:** Atika Ailli, Nadia Handaq, Hanane Touijer, Aman Allah Gourich, Aziz Drioiche, Khalid Zibouh, Brahim Eddamsyry, Fadoua El Makhoukhi, Aicha Mouradi, Yousef A. Bin Jardan, Mohammed Bourhia, Abdelhakim Elomri, Touriya Zair

**Affiliations:** 1 Research Team of Chemistry of Bioactive Molecules and the Environment, Laboratory of Innovative Materials and Biotechnology of Natural Resources, Faculty of Sciences, Moulay Ismaïl University, B.P.11201 Zitoune, Meknes 50070, Morocco; 2 Laboratory of Biology, Environmental and Sustainable Development, Hight Normal School, Abdelmalek Essaadi University, Tetouan 93000, Morocco; 3 Department of Pharmaceutics, College of Pharmacy, King Saud University, Riyadh 11451, Saudi Arabia; 4 Department of Chemistry and Biochemistry, Faculty of Medicine and Pharmacy, Ibn Zohr University, Laaoune 70000, Morocco; 5 University of Rouen Normandy, INSA Rouen Normandy and CNRS, Laboratory of Organic, Bioorganic Chemistry, Reactivity and analysis (COBRA-UMR 6014), 76000 Rouen, France

**Keywords:** plants, chemical composition, antioxidant activity, antimicrobial activity, correlation

## Abstract

In this work, the chemical composition and antioxidant and antimicrobial activities of the essential oils (EOs) of six species—*Laurus nobilis*, *Chamaemelum nobile*, *Citrus aurantium*, *Pistacia lentiscus*, *Cedrus atlantica*, and *Rosa damascena*—have been studied. Phytochemical screening of these plants revealed the presence of primary metabolites, namely, lipids, proteins, reducing sugars, and polysaccharides, and also secondary metabolites such as tannins, flavonoids, and mucilages. The essential oils were extracted by hydrodistillation in a Clevenger-type apparatus. The yields are between 0.06 and 4.78% (mL/100 g). The analysis of the chemical composition carried out by GC-MS showed the presence of 30 to 35 compounds and represent between 99.97% and 100% of the total composition of EOs, with a variation in the chemical composition detected at the level of the majority compounds between these species. Indeed, in the EO of *Laurus nobilis*, 1,8-cineole (36.58%) is the major component. In *Chamaemelum nobile* EO, the most abundant compound is angelylangelate (41.79%). The EO of *Citrus aurantium* is rich in linalool (29.01%). The EO of *Pistacia lentiscus* is dominated by 3-methylpentylangelate (27.83%). The main compound of *Cedrus atlantica* is β-himachalene (40.19%), while the EO of *Rosa damascenaa* flowers is rich in n-nonadecane (44.89%). The analysis of the similarity between the EOs of the plants studied by ACH and ACP showed that the chemical composition of the EOs makes it possible to separate these plants into three groups: the first represented by *Chamaemelum nobile*, because it is rich in oxygenated monoterpenes, the second defined *Cedrus atlantica* and *Rosa damascena*, which are rich in sesquiterpenes, and the third gathers *Pistacia lentiscus*, *Laurus nobilis* and *Citrus aurantium*, which are composed of oxygenated sesquiterpenes and monoterpenes (these three species are very close). The study of the antioxidant activity showed that all the EOs tested have a high capacity for scavenging free radicals from DPPH. The EOs of *Laurus nobilis* and *Pistacia lentiscus* showed the highest activity, 76.84% and 71.53%, respectively, followed by *Cedrus atlantica* EO (62.38%) and *Chamaemelum nobile* (47.98%) then *Citrus aurantium* EO (14.70%). Antimicrobial activity EO was tested against eight bacterial strains and eight fungal strains; the results showed that EOs exhibit significant bactericidal and fungicidal activities against all the microorganisms tested, of which the MICs of the bacterial strains start with 5 mg/mL, while the MICs of the fungal strains are between 0.60 mg/mL and 5 mg/mL. Thus, these EOs rich in antimicrobial and antioxidant components can serve as a natural alternative; this confirms their use as additives in cosmetics.

## 1. Introduction

Aromatic and medicinal plants are precious reservoirs of bioactive molecules; they are able to produce diverse natural substances [1]. In fact, there are two distinct metabolisms that distinguish aromatic and medicinal plants. Proteins, carbohydrates, and lipids are produced by primary metabolism, while terpenoids, alkaloids, and polyphenols are produced by secondary metabolism. Terpenoids, alkaloids, and polyphenols are particularly important in the pharmaceutical, cosmetic, and agri-food industries [2].

Essential oils (EOs) or plant essences are part of the terpenoids, and their chemical composition is quite complex; it is a mixture of various molecules, including in particular, terpenes (non-aromatic hydrocarbons) and oxygenated compounds (alcohol, aldehyde, ketone, and ester) [3]. The time of harvest, the location of cultivation, the part of the plant used, and the method of production are just a few examples of the many variables that can affect the chemical composition and quality of EOs extracted from different plant species [4]. They can also be modified during extraction or during storage [5]. Plant EOs are created and released by specific cells or organs, where they remain confined [6]. All of the plant’s organs have the capacity to store them. These unique histological structures vary across botanical families and are found on or near the surface of the plant [7].

Currently, scientific research is focused on creating new applications and taking advantage of the qualities of EOs in many industries, including the food, cosmetics, and pharmaceutical industries [8]. They are frequently used as additives in the cosmetic industry because they have many advantages. Their biological effects include stimulant, diuretic, analgesic, antiseptic, and antimicrobial properties [9]. Because the fatty acids, fatty oils, and surfactants used in the formulation of cosmetic products frequently emit an unpleasant odor, their main use in the cosmetic industry is due to their pleasant aromas [10]. Morocco offers a rich and diverse floristic biodiversity as a result of its varied climatic and ecological conditions [11]. The gradual discovery of the uses for their essential oils (EOs) in healthcare and other areas of economic interest, as well as for their anti-inflammatory, antiseptic, antiviral, antimicrobial, and antitoxic properties, has been particularly advantageous for aromatic and medicinal plants [9].

The Moroccan plant is mainly Mediterranean (38% of the total of the genera), 30% of the metropolis, 22% of the Nordic, 9% of Africa, and 19% endemic, including 5% endemic to the Mediterranean [11]. Among the aromatic and medicinal plants of Morocco, we chose six plants, namely, *Laurus nobilis*, *Chamaemelum nobile*, *Citrus aurantium*, *Pistacia lentiscus*, *Cedrus atlantica*, and *Rosa damascena*. These species are increasingly exploited locally and in demand on the international market, and in particular by the cosmetic industry.

The *Laurus nobilis* is a species of evergreen shrub of the Lauraceae family. It is native to the Mediterranean basin [12]. *Laurus nobilis* EO is currently used in herbal medicine for the treatment of several neurological, dermatological, urological, rheumatic, and dermatitis problems [13].

*Chamaemelum nobile* in the Asteraceae family is a low-growing perennial that forms a spreading carpet of aromatic foliage [14]. This plant is usually found in dry fields and around gardens and cultivated land. Many previous studies have demonstrated that this plant’s anti-inflammatory, antimicrobial, and antiseptic properties make it a common component of traditional medicine. Additionally, it has long been used to treat a number of ailments such as malaria, peptic ulcers, and wound healing [5].

*Citrus aurantium* is a species of tree in the Rutaceae family [15]. The fragrant leaves, twigs, and flowers have many food and perfumery applications. In addition, the field of application of this species is widened in the treatment of gastrointestinal diseases and obesity [16]. *Citrus aurantium* EO is considered a source of bioactive compounds with antimicrobial properties [17], antioxidants [18], anti-inflammatories [19], and anti-anxiety effects [20].

*Pistacia lentiscus* is part of the Anacardiaceae family, native to the Middle East and the Mediterranean [21]. This species is of great interest for traditional and pharmaceutical medicine. The EO of this plant has attracted the interest of researchers due to its antioxidant potential and antimicrobial and anti-inflammatory activity [22].

*Cedrus atlantica* is a species of coniferous tree in the Pinaceae family [23].The EO isolated from this species is considered as a natural substance and is used as a raw material for the production of other fragrance chemicals, as flavoring additives for food and beverages, as perfuming agents in a variety of household products, and as flavoring additives for cosmetics. Additionally, this EO’s biological properties, including its antioxidant, antimicrobial, antiviral, anti-inflammatory, analgesic, and anticancer properties, have been reported in earlier studies [4,23,24].

*Rosa damascena* is a species of the Rosaceae family, commonly called Damask rose or oleaginous rose. It is one of the most important aromatic plants in the world. In Morocco, this plant is widely grown. It can be used as a food additive in addition to being an ornamental plant in gardens and parks. The industries of perfumery, cosmetics, and pharmaceuticals use its byproducts, including rose essential oil, rose water, rose concrete, and rose absolute, as their primary products [25].

The main objective of this work is, on the one hand, the contribution to the phytochemical study and characterization of the chemical families present in the EOs of *Laurus nobilis*, *Chamaemelum nobile*, *Citrus aurantium*, *Pistacia lentiscus*, *Cedrus atlantica*, and *Rosa damascena* harvested from different regions of Morocco. On the other hand, it is the evaluation of the antimicrobial and antioxidant activities of the EOs. This research is done in order to value potentially commercially viable Moroccan aromatic and medicinal plants as well as for cosmetics industry research.

## 2. Results and Discussion

### 2.1. Quality Control of Plant Material

The samples were subjected to quality control by measuring their moisture content (MC), pH, acidity, ash content, and heavy metal content, among other distinctive parameters. The results are shown in Table 1.

#### 2.1.1. Moisture Content

The moisture content of the plants studied varies between 10.29% and 83.61% (Table 1). The highest content is recorded for *Citrus aurantium* (83.61%), while low moisture levels are recorded for *Chamaemelum nobile* (18.01%), *Laurus nobilis* (14.88%), and *Cedrus atlantica* (13.33%), followed by *Rosa damascena* and *Pistacia lentiscus* having the rates (10.29%) and (10.27%), respectively. The high value of the moisture content of *Citrus aurantium* can be explained by the use of fresh plant material.

#### 2.1.2. pH Determination

The quality of plant matter is judged by its pH, which also determines how well plants can be preserved; one of the main challenges facing microbial flora is overcoming it to ensure its proliferation. In our situation (Table 1), the pH of the species under study ranges from 4.03 to 5.76. With the availability of mineral elements, these values give these plants a good character quality.

#### 2.1.3. Ash Content

The amount of ash (mineral matter) of a sample reveals the mineral content of inorganic noncombustible material it possess. The studied species have comparable levels of organic matter (Table 1); they vary between 71.86% and 96.33%. On the other hand, the ash content is low, varying between 3.67% and 28.14%.

#### 2.1.4. Heavy Metal Assays

During growth, development, and processing, medicinal plants are easily contaminated. The heavy metals present in plants are typically ingested by people after being collected and transformed. These heavy metals can interfere with the normal operation of the central nervous system, liver, lungs, heart, kidneys, and brain and cause hypertension, stomach pain, skin rashes, intestinal ulcers, and various cancers. To remedy this, we opted to verify the presence of heavy metals in the species studied. The dosage of heavy metals in the six plants studied has been the subject of very little work. In our case, a total of six elements, namely, Chromium (Cr), Antimony (Sb), Lead (Pb), Cadmium (Cd), Iron (Fe), and Titanium (Ti), were determined for the six plants by atomic absorption spectrophotometry. Table 2 shows very low and variable concentrations of the metals; others are undetectable. These variations depend on the assimilated element and the species. Additionally, values from the tested samples were lower than the FAO/WHO-mandated limit value.

### 2.2. Phytochemical Screening of Plant Material

Phytochemical screening is a qualitative analysis whose purpose is the characterization of groups of chemical families in a plant. Its principle is based on the formation of insoluble complexes via precipitation reactions or colored complexes via coloring reactions.

#### 2.2.1. Primary Metabolites

The screening of primary metabolites allowed us to highlight the presence of lipids, proteins, and reducing sugars in *Laurus nobilis*, *Chamaemelum nobile*, and *Citrus aurantium*, while *Pistacia lentiscus* is devoid of polysaccharides. The results also showed an absence of lipids and reducing sugars and a low protein and polysaccharide content for *Rosa damascena* (Table 3).

#### 2.2.2. Secondary Metabolites

The phytochemical tests described by Harborne (1998) [26] to detect secondary metabolites are also based on color changes or the formation of precipitates using specific reagents. Table 4 groups together all the results of the phytochemical screening of secondary metabolites obtained for the six plants studied. These results therefore showed the presence of total tannins and flavonoids in all the samples. The tannins catechists are present in all samples except *Cedrus atlantica*, while gallic tannins are absent in *Chamaemelum nobile* and *Cedrus atlantica*. The cyanidin reaction showed a positive result for *Laurus nobilis* and *Rosa damascena*. Anthocyanins are present in *Pistacia lentiscus* and *Rosa damascena*. Moreover, a total absence of alkaloids was observed in all the species studied. While mucilages are absent only in *Chamaemelum nobile* and *Cedrus atlantica*, a weak presence of saponosides was observed only in *Laurus nobilis*, *Pistacia lentiscus*, and *Rosa damascena*. In addition, flavonoids possess anti-inflammatory, antiviral, antispasmodic, and antibacterial activities; tannins are antidiarrheal and antiseptic; sterols and triterpenes have antiviral, analgesic, and anti-inflammatory properties; and saponosides are anti-inflammatory, antifungal and antiviral [27].

### 2.3. Yields and Quality Control of Essential Oils

#### 2.3.1. Yields of EOs

**Figure 1:**
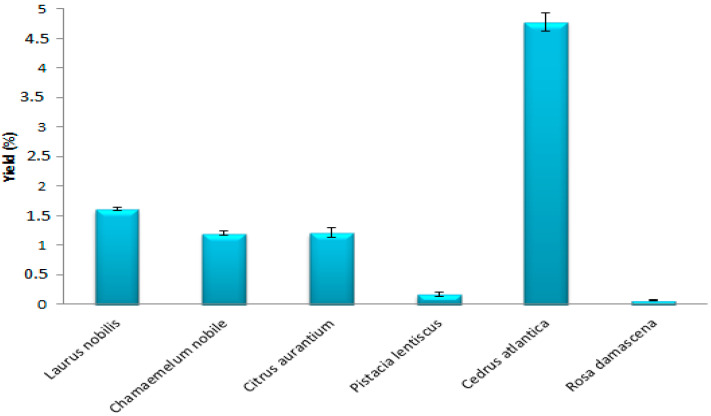
Yields of EOs of the plant species.

The results obtained showed that the yields of EO, extracted by hydrodistillation of the species studied, vary between 0.06 and 4.78% (Figure 1). The best EO yield obtained is that of *Cedrus atlantica* (4.78%), followed by *Laurus nobilis* (1.61%), *Citrus aurantium* (1.21%), and *Chamaemelum nobile* (1.20%). However, the yield of *Pistacia lentiscus* and *Rosa damascena* are low (0.17% and 0.06%). Comparing these results with previous studies, we noticed that Belkacem et al. (2021) [24] and Paun et al. (2013) [28] found lower yields than ours of *Cedrus atlantica* EO (0.41% and 0.62%, respectively). Moreover, Abdoul-Latif et al. (2021) [5] obtained an EO yield of around 0.44% for *Chamaemelum nobile*, while other researchers have found superior yields, such as Milia et al. (2020) [29] who obtained a higher yield (0.41%) of EO from *Pistacia lentiscus*. Variations in EO levels in plants may be due to several factors, in particular the collection region, the climate, the altitude, and the type of soil [30].

#### 2.3.2. Chemical Composition of EOs of the Plants Studied

The GC–MS analysis of the EOs of the six plants studied showed a variation in the chemical composition in terms of quality and quantity. The chromatographic profiles (Supplement File) made it possible to identify between 31 and 35 compounds. The identification of the chemical composition of the EO of the leaves of *Laurus nobilis* revealed the presence of 35 chemical compounds, representing approximately 99.98% of the total composition, including 13 compounds with percentages greater than 1% (Table 5, Supplement File). 1,8-cineole (36.58%), which is the main component of this EO, is considered as a medicinal and phenological treatment of laurel, followed by α-terpinylacetate (15.42%), sabinene (12.08%), methyleugenol (5.34%), α-pinene (5.29%), β-pinene (4.06%), linalool (3.45%), and α-terpineol (3.15%). We also found the presence of three chemical families with high levels, namely, oxygenated monoterpenes (47.52%), monoterpenes (27.39%), and oxygenated sesquiterpenes (23.81%), while sesquiterpenes are present with lower percentages (1.26%). This comparison agrees with other studies carried out in Mediterranean areas. Indeed, Caputo et al. (2017) [12] showed that 1,8-cineole (31.9%), sabinene (12.2%), and linalool (10.2%) are the main components of EO in bay leaves, while other components such as α-terpinyl acetate (5.9%), α-pinene (5.8%), α-terpineol (3.3%), and methyl-eugenol (3.3%) are only present with lower percentages. The percentage of 1,8-cineole (36.58%) is lower than the values noted in previous works: Fidan et al. (2019) [31] (41.0%), Ekren et al. (2013) [32] (44.97%), and Snuossi et al. (2016) [33] (56.0%). However, the sabinene content was 12.08%, higher than that of the EOs analyzed by Derwich et al. (2009) [34], Snuossi et al. (2016) [33], and Yalcin et al. (2007) [35], who found sabinene percentages of 6.13, 3.5, and 6.13%, respectively.

In the EO of *Chamaemelum nobile* flowers, 33 volatile constituents, representing 99.56% of the total composition, were identified, ten compounds among them exceeding 1% (Table 6, Supplement File). The most abundant constituent was angelylangelate (41.79%), followed by methallylangelate (19.39%) and 2-hydroxy-2-methyl-3-butenyl angelate (19.33%). Carvacrol presents an amount of 2.15%, while the other compounds represented only small percentages: (3*Z*)-hexenyltiglate (1.83%), isobutylangelate (1.46%), 2-methylbut-2-en-1-yl isobutyrate (1.36%), and 2-methylallyl isobutyrate (1.01%). The results also show that of these 33 constituents, 94.32% belong to the family of oxygenated monoterpenes, while the oxygenated sesquiterpenes do not exceed 5.04%. A different chemical composition has been noticed in previous studies. Abdoul-Latif et al. (2021) [5] reported that the *Chamaemelum nobile* EO collected from the Khenifra-Morocco region is characterized by verbenone (33.74%), pulegone (26.45%), and 3-cyclohexene-1-one, 2- isopropyl-5-methyl- (8.90%). Roozbeh and Dong-Jin (2017) [36] reported that EO from the same plant collected in Iran is characterized by Chamazulene (31.12%), β-Pinene (10.11%), ά-bisabolol (7.32%), a pigenin-7-glucoside (6.20%), ά-bisabololoxide A (5.98%), α-pinene (5.97%), and β–thujone (4.84%). Aremu et al. (2018) [37] also showed a difference in the chemical composition of *Chamaemelum nobile* EO compared to our study, with alpha-bisabolol being the most abundant quantitatively (>50%). The second most abundant compound was farnesene (5.35%), followed by spathulenol (2.56%).

From the EO of *Citrus aurantium* flowers, 34 compounds were identified, representing 99.81% of the total identified (Table 7, Supplement File). The main volatile compounds in the EO from the flowers of *Citrus aurantium* are linalool (29.01%), (2*E*,6*E*)-farnesol (12.77%), linaloolacetate (12.46%), and (2*E*,6*E*)-farnesol (12.77%). This essential oil also contains other compounds such as (*E*)-Nerolidol (6.91%), Limonene (6.42%), (*E*)-β-Ocimene (5.93%), α-Terpineol (5.47%), and Geranylacetate (4.57%). These compounds are divided into four chemical classes: oxygenated monoterpenes (38.9%), oxygenated sesquiterpenes (39.69%), monoterpenes (19.30%), and the sesquiterpenes (1.45%). On the other hand, a different composition was obtained by Kacániová et al. (2020) [15], who showed the presence of linalyl acetate (63.37%), -terpineol (8.84%), geranyl acetate (6.02%), and neryl acetate (3.77%). In addition, Bourgou et al. (2012) [38] reported that the main volatile compound obtained in *Citrus aurantium* fruits was limonene, with a rate of 90.95%, 69.71%, and 69% in orange EO bitter, lemon, and tangerine, respectively. Sarrou et al. (2013) [39] worked on the EO of the skin of *Citrus auratium* grown in Greece; they also declared the abundance of limonene (94.67%), followed by β-myrcene (2%), linalool (0.67%), α-pinene (0.62%), and β-pinene (0.53%).

In the EO of *Pistacia lentiscus* leaves, 32 compounds were identified, representing 100% of the total EO (Table 8, Supplement File). The results show that the oil obtained was dominated by 3-methylpentylangelate (27.83%), isobutylangelate (10.65%), α-pinene (10.16%), and methallylangelate (8.4%). Moreover, these 32 compounds are divided into four chemical classes: oxygenated monoterpenes (49.35%), oxygenated sesquiterpenes (28.71%), monoterpenes (17.50%), and sesquiterpenes with a low percentage (4.44%). Studies carried out on *Pistacia lentiscus* from Turkey have shown different compositions. Indeed, the results obtained by Duru et al. (2003) [40] showed the EO richness of *Pistacia lentiscus* leaves in β-pinene (38.7%) and α-pinene (21.7%). Other compounds with lower percentages were identified, among others, pinocarvone (5.3%), α-ylangene (4.0%), limonene (3.8%), and n-nonanol (3.5%). Tabanca et al. (2020) [21] showed the abundance of α-pinene (56.2%), myrcene (20.1%), and β-pinene (3.1%).

EO analysis of *Cedrus atlantica* wood allowed the identification of 31 compounds, representing 99.97% of the total composition (Table 9). The main components of this oil are β-himachalene (40.19%), α-himachalene (15.74%), γ-imachalene (10.49%), and cis-thujopsenal (4.57%). The compounds identified are classified into three chemical families, namely, sesquiterpenes (81.16%), oxygenated sesquiterpenes (18.35%), and monoterpenes (0.46%). On the other hand, Belkacem et al. (2021) [24] showed that the EO of *Cedrus atlantica* wood collected from Adekar, Algeria, is characterized by monoterpene hydrocarbons (89.18%). Sesquiterpenes were detected with lower levels. Chemical analysis also revealed that our sample had different constituents compared to those analyzed by Aberchane et al. (2004) [41]; they found the abundance of β-Himachalene (14.62%), α-(*E*)-Atlantone (30.75%), Himachalol (6.50%), and α-Himachalene (5.32%). On the other hand, the study carried out by Boudarene et al. (2004) [42] on the EO in the seeds of this plant also showed different constituents to our findings, whose main components found were α-pinene (37.1%), β-pinene (8.6%), myrcene (3.6%), limonene (2.5%), bornyl acetate (5.4%), β-farnesene (6.8%), and manool (20.7%).

GC–MS analysis of essential oil extracted from *Rosa damascenaa* flowers revealed the presence of 31 compounds, representing 98.88% of the total composition (Table 10). The majority compound is n-nonadecane (44.89%); in addition, there are other compounds with significant percentages, namely, n-heneicosane (19.49%), 1-nonadecene (12.98%), n-heptadecane (5.35%), and n-tetracosane (4.04%), while the percentages of the remaining compounds are relatively low (<1%). As for the chemical families, the sesquiterpenes represent 94.02%; however, the oxygenated sesquiterpenes did not exceed 3.15%, while the oxygenated monoterpenes represented only 1.71%. The work published by Mansureh Ghavam et al. (2021) [43], carried out on the EO of *Rosa damascena* collected in different regions of Iran, showed relatively different compositions compared to our results. The main compounds for all the plants harvested are citronellol (36.70–9.18%), geraniol (12.82–0.47%), nonadecane (22.73–10.36%), l heneicosan (31.7–11.43%), and 1-nonadecene (6.03–3.93%).

Overall, the constituents of these essential oils are mixtures of complex molecules that have interesting properties in several industries, particularly pharmaceuticals and cosmetics [9].

**Figure 2:**
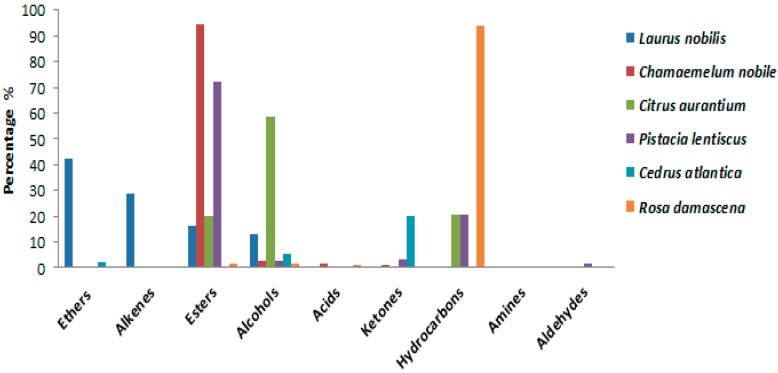
Chemical families identified in the EO_S_ of plants species (%).

The compounds identified in each plant are found in highly variable percentages (Figure 2). Indeed, *Laurus nobilis* oil is rich in ethers (42.33%), followed by alkenes (28.65%), while *Chamoemelum nobile* is characterized by a very high content of esters (94.29%). *Citrus auraium* is composed mainly of alcohols (58.61%), followed by hydrocarbons (20.75%) and esters (20.16%). Furthermore, *Pistacia lentiscus* is rich in esters (72.18%), and the percentage of hydrocarbons is 20.72%. *Cedrus atlantica* is rich in hydrocarbons (68.06%); it also contains a significant percentage of ketones (19.81%). On the other hand, *Rosa damascena* is essentially composed of hydrocarbons (94.02%) (Table 11). In addition, we found other chemical families in the essential oils of these plants but with low levels, namely, alcohols, amines, aldehydes, and acids. With a change in the chemical composition of the species under study, these qualitative and quantitative variations in the chemical composition of the EOs studied actually allowed us to confirm an important interspecific chemical polymorphism in these EOs. At the level of the majority of compounds, this modification is remarkable. Phytochemical expression in plants is largely influenced by the plant–environment interaction, according to research published in the literature [44,45].

### 2.4. Analysis of the Similarity between the EOs of the Plants Studied

#### 2.4.1. Hierarchical Ascending Classification (HAC)

**Figure 3:**
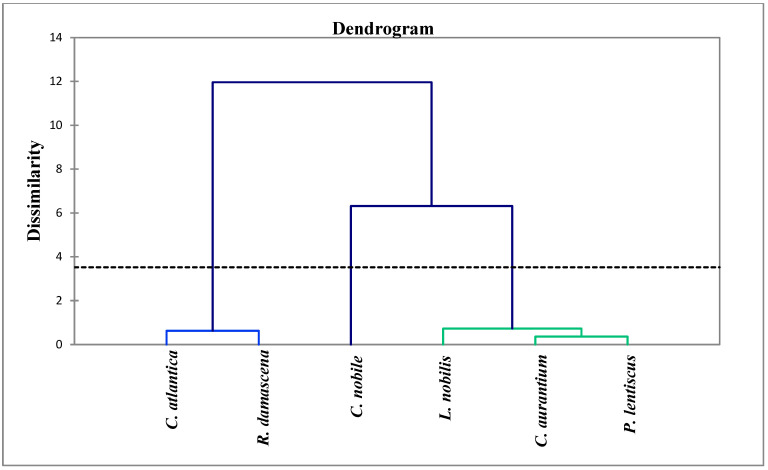
Phylogenetic tree of EOs of the plants studied.

The analysis of the chemical composition of the six plants studied showed diversity between the compounds that characterize each plant. Indeed, an analysis of the similarity between the chemical compositions of these EOs was carried out using the ascending hierarchical classification to create groups of uniform observations from the samples, each group being clearly distinguished from the others. After that, a dendrogram or tree classification was created using the samples from the analysis. The variation between the studied plants’ Eos is depicted in Figure 3. The results showed that the samples can be grouped into three distinct groups according to the distance that separates them. The first group (I) includes the EO of *Cedrus atlantica* and *Rosa damascena*, the second group (II) includes the EO of *Chamaemelum nobile*, and the third group (III) includes the EO of three species, namely, *Citrus aurantium*, *Laurus nobilis*, and *Pistacia lentiscus*. The dendrogram analysis also showed that the species of group I have 12% similarity with the other groups. Similarly, group II has 6.5% similarity with group III; the latter includes three samples of EO which are heterogeneous. *Laurus nobilis* is alone in a class, and it has a similarity of 0.9% with the species *Citrus aurantium* and *Pistacia lentiscus*; the fact that these two species are grouped together within the same class indicates that there is intra-class homogeneity. The close proximity of the two species to one another explains why they are so similar at the level of the chemical profile.

#### 2.4.2. Principal Component Analysis (PCA)

**Figure 4:**
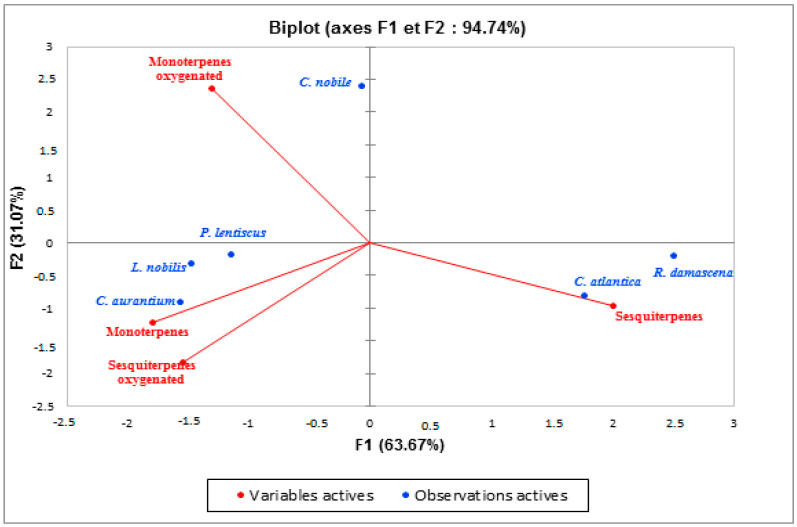
Principal component analysis carried out on the main chemical families of EOs of the species studied.

In order to identify similarities and differences between samples, principal component analysis is a technique that breaks down data by examining the relationships between all variables. The distribution of the species studied in the plane formed by two main axes (F1 and F2) explains 94.89% of the variability (Figure 4). This analysis showed a significant difference in the percentage of chemical families found. Indeed, we noticed the heterogeneity in the chemical compositions of the essential oils of these plants. The EO samples could be divided into three distinct subgroups: the first subgroup represented by *Chamaemelum nobile*, the second subgroup defined by *Cedrus atlantica* and *Rosa damascena*, while the third subgroup included *Pistacia lentiscus*, *Laurus nobilis*, and *Citrus aurantium*. The F1 axis explained 50.62% of the total variability; *Cedrus atlantica* is designed on this axis on the positive side (this zone is characterized by a high content of sesquiterpenes), while *Pistacia lentiscus*, *Laurus nobilis*, and *Citrus aurantium* were designed in F1 negative. In addition, F2 explained 44.27% of the total variability. Figure 4 demonstrated that *Chamaemelum nobile* is projected at positive F2. This zone is characterized by a high percentage of oxygenated monoterpenes. While *Pistacia lentiscus*, *Laurus nobilis*, and *Citrus aurantium* are negatively associated with F2, this group has a similar chemical composition, i.e., similar amounts of monoterpenes and oxygenated sesquiterpenes. These data allowed us to conclude that the chemical composition of the essential oils of *Chamaemelum nobile* and *Cedrus atlantica* is different, while *Pistacia lentiscus*, *Laurus nobilis*, and *Citrus aurantium* are very close to each other.

### 2.5. EO Quality Control

The physicochemical properties such as acid, peroxide, iodine, and saponification values constitute the means of verification and quality control of the essential oil. These measurements are determined according to the specific protocols. Thus, the results are reported in Table 12 and summarized below.

#### 2.5.1. Density

The density of essential oils extracted from the plants studied varies between 0.873 and 0.937 g/mL (Table 12). Indeed, the density of EO from *Cedrus atlantica* (0.937 g/mL), *Chamaemelum nobile* (0.921 g/mL), and *Laurus nobilis* (0.912 g/mL) was slightly higher than that of EO from *Pistacia lentiscus* (0.878 g/mL), *Citrus aurantium* (0.878 g/mL), and Rosa damask (0.878 g/mL). In addition, we note that the EOs of the latter have the same density (0.878 g/mL). Additionally, numerous studies have demonstrated the impact of the vegetative cycle, the type of plant (fresh or dried), the time of harvest, and the method of extraction on the quality of the essential oil [46,47,48].

#### 2.5.2. Acid Value

The acid value provides information on the content of free fatty acids in the EO. A low acidity value characterizes the purity and stability of the oil at room temperature [49]. The EOs of *Laurus nobilis* and *Citrus aurantium* have lower acid numbers, 0.5 and 0.56, respectively, followed by the EO of *Pistacia lentiscus* (1.12) and *Cedrus atlantica* (1.68), while the highest acidity value is recorded in the essential oil of *Chamaemelum nobile* (2.24) (Table 12).

#### 2.5.3. Iodine Value

The iodine value is a chemical parameter that provides information on the degree of the overall establishment of the oil. The more unsaturated an oil, the higher its iodine value [50]. The values of the iodine indices of the essential oils of the species studied are between 0.812 and 1.1. The lowest iodine indices correspond to the oils of *Laurus nobilis* (0.812) and *Pistacia lentiscus* (0.888), followed by *Cedrus atlantica* (0.939), *Chamaemelum nobile* (0.989), and *Citrus aurantium* (1.1) (Table 12). These values obtained are lower than those provided by the Codex Alimentarius standard (87–111 g of iodine/100 g). Moreover, these values can be explained by a possible oxidation of unsaturated fatty acids [51]. The conservation of these oils could be done without too much risk of auto-oxidation.

#### 2.5.4. Peroxide Value

The peroxide index is a good indicator of the state of conservation of a fatty substance. This is a very useful criterion for assessing the first stages of the oxidative deterioration of oil [51,52]. The value of the highest peroxide is noted by *Cedrus atlantica* (170); this index is higher than the essential oil of *Pistacia lentiscus* (64), *Citrus aurantium* (40), and *Chamaemelum nobile* (30). The lowest peroxide index is obtained for *Laurus nobilis* EO (4) (Table 12). By comparing our results with those of the commercial standards, CODEX STAN 210-1999, we find that all the EOs analyzed comply with the standards, which allows us to classify them as good quality.

#### 2.5.5. Saponification Value

The determination of the saponification index allows us to characterize the molecular weight and the average length of the fatty chains to which it is inversely proportional [52]. The saponification values obtained are very low and almost similar for all the EOs studied, between 0.418 and 0.603 (Table 12). This explains the richness of EOs in short chains of fatty acids [52].

### 2.6. Antioxidant Activity of EOs

**Figure 5:**
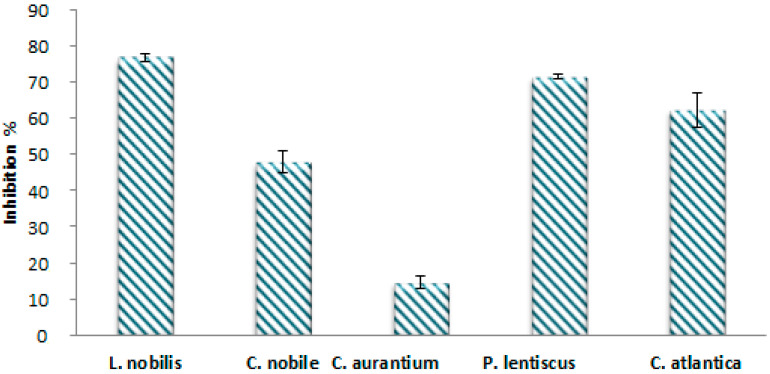
Percentage of inhibition of DPPH° by EOs of plants species.

In the present study, the essential oils extracted from the studied plants were tested for their antioxidant activity by the DPPH method (2,2-diphenyl-1-picrylhydrazyl). The results are shown in Figure 5. The DPPH radical scavenging capacity was evaluated in terms of the percentage of inhibition of DPPH free radicals by the antioxidants in the different EOs extracted. A higher inhibition percentage reflects better antioxidant activity. As a result, all EOs tested showed a high capacity to scavenge these radicals. *Laurus nobilis* and *Pistacia lentiscus* EOs showed the highest antioxidant activity, with inhibition percentages of 76.84% and 71.53%, respectively. In addition, a powerful antioxidant power was also recorded for *Cedrus atlantica* EO (62.38%), followed by *Chamaemelum nobile* with an inhibition percentage of 47.98%, while the lowest activity (14.70%) was obtained with *Citrus aurantium* EO.

The evaluation of antioxidant activity using the DPPH test revealed a variable antioxidant response from one species to another. In addition, the ability of these oils to scavenge the DPPH radical is very important. This interesting biological activity can be attributed to the chemical composition of EOs [24]. In fact, these essential oils’ constituents are intricate blends of hydrocarbons, alcohols, aldehydes, ketones, and esters, and they are distinguished by the predominance of terpene products. Oils that have high percentages of monoterpenes and oxygenated monoterpenes—*Laurus nobilis*, *Pistacia lentiscus*, and *Cedrus atlantica*—have shown high DPPH scavenging efficiency. These results agree with those of other authors, in particular [33,53,54]. In addition, studies show that essential oils that contain oxygenated monoterpenes and/or sesquiterpenes have more antioxidant properties. The dominance of 1,8-cineole is also an important factor in the activity of bay EO [54], but there might also be other minor compounds that work antagonistically or in a synergistic way to combat free radicals [24,53,55].

### 2.7. Antimicrobial Activity

#### 2.7.1. Antibacterial Activity

Eight bacterial strains that belonged to both Gram-positive and Gram-negative bacteria were subjected to various concentrations of the EOs of the plants under study to test their antibacterial activity (Table 13). The results showed that these essential oils exhibit significant bacteriocidal activities against all the bacteria tested. Indeed, the oils of all the plants—*Laurus nobilis*, *Chamaemelum nobile*, *Citrus aurantium*, *Pistacia lentiscus*, and *Cedrus atlantica*—have shown very high efficiencies; they inhibit the growth of the strains *Staphylococcus epidermidis*, *Proteus mirabilis*, *Staphylococcus aureus* BLACT, *Escherichia coli* ESBL, *Enterobactercloacae*, *Pseudomonas aeruginosa*, *Klebsiella pneumonia*, and *Pseudomonas aeruginosa* from 5 mg/mL. These results are particularly interesting in relation to the antibiotics Gentamycin, Amoxicillin–Clavulanate, Vancomycin, and Trimethoprim–Sulfamethoxazole (Table 14). Therefore, the results mentioned above demonstrated that EOs from these plants can serve as a natural antibacterial alternative.

#### 2.7.2. Antifungal Activity

The antifungal activity of EOs was also tested against eight fungal strains, yeasts and molds. The results in Table 15 showed that all the strains studied exhibit sensitivity with almost similar degrees with respect to the essential oils of the plants tested. The MICs of these Eos are found from 5 mg/mL against all strains except *Candida parapsilosis*, which is inhibited by a very low concentration of EOs from *Pistacia lentiscus* and *Cedrus atlantica* (MIC = 0.6 mg/mL), followed by EOs of *Laurus nobilis*, *Chamaemelum nobile*, and *Citrus aurantium* (MIC = 1.2 g/mL) (Table 15).

### 2.8. Correlation of the Chemical Composition of EOs and Their Antimicrobial Activities

**Figure 6:**
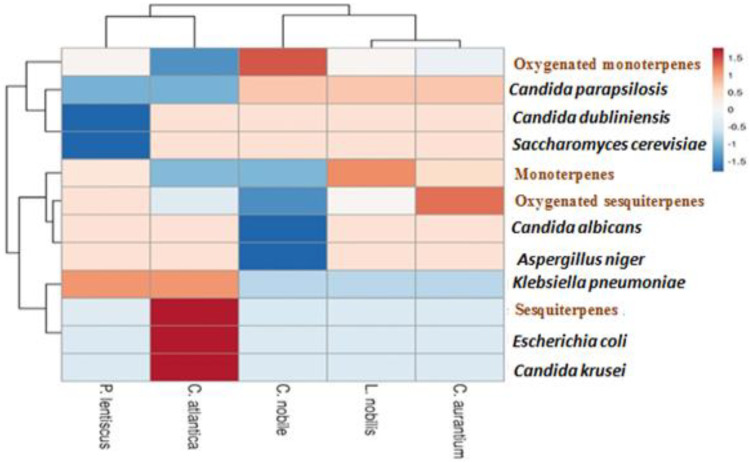
Correlation between the chemical composition of essential oils and their antimicrobial activities.

The heat map is a very popular representation, especially in various sciences and among the graphical representations commonly used to determine the effect of phenomena including those organic [56,57]. The heat map draws attention to the quantitative values in a rectangular data matrix. The color pattern changes depending on the quantitative value of the corresponding data matrix element, starting with blue for the lowest value and gradually increasing in intensity to red for the highest value. Here, the heat map was constructed using EO chemical composition data to determine the correlation between this composition and antimicrobial activity (Figure 6). This correlation showed that oxygenated monoterpenes had a positive and greater effect on yeast *Candida parapsilosis* suivi of yeasts *Candida dubliniensis* and *Saccharomyces cerevisiae*. Oxygenated sesquiterpenes are more effective against the bacteria *Klebsiella pneumoniae*. Likewise, they have a moderate effect against the fungus *Aspergillus niger* and the yeast *Candida albican*. In addition, the sesquiterpenes present in *Cedrus atlantica* have a very strong positive correlation with respect to the inhibition of strain growth in wild *Escherichia coli* and *Candida krusei*. This study showed that the EOs of the plants studied are effective against the microbial strains tested with varying degrees.

In this study, results showed that each EO comprises a mixture of a number of constituents that may have a broad spectrum of antimicrobial activity. The main constituent of bay EO in our results and in some previous studies was 1,8-cineole; this compound showed antimicrobial activity against several strains of the microorganisms [12,31,57]. Additionally, the effectiveness of bay EO in this study in inhibiting microbial growth was likely due to the synergistic or antagonistic interaction of 1,8-cineole with the oxygenated terpenes of the oil [57,58]. For the EO of *Chamaemelum nobile*, our results agree with previous studies, in particular by Abdoul-Latif et al. (2021) [5], that showed that *Chamaemelum nobile* EO revealed significant antibacterial activity against several strains, namely, *Staphylococcus epidermidis* (CECT 231), *Listeria monocytogenes* (CECT 934), *Listeria innocua* (CECT 910), *Escherichia coli* (CECT 515), *Yersinia enterocolitica* (CECT 4315), and *Pseudomonas aeruginosa* (CECT 108), especially with bacteriocidal effects. In addition, Ansari Dezfooli et al. (2012) [59] also noted a strong inhibition of the growth of the *Psedomonas tolaasii* strain by *Chamaemelum nobile* EO, and they linked this activity to the presence of α-bisabolol oxide (58.8%) as the majority component. The chemical composition of *Citrus aurantium* EO is dominated by 58.61% alcohols. Alcohols are particularly effective against microbial strains because they can dissolve in aqueous media and severely damage microorganism’s cell walls [48]. Alcohol has bacteriocidal activity as opposed to bacteriostatic activity [59]. Similarly, the antibacterial effect of *Citrus aurantium* EO is also reported in previous studies. Ben Hsouna et al. (2017) [60] showed that this EO has moderate to strong antimicrobial activity against bacterial and fungal species due to the synergistic effect of the majority compounds, namely, limonene (27.5%), E-nerolidol (17.5%), α-terpineol (14%), and α-terpinylacetate (11.7%). *Pistacia lentiscus* EO exhibited antimicrobial activity against all microorganisms tested. These results may suggest a synergy existing between the oxygenated monoterpene fractions, in particular, 3-Methylpentylangelate, α-pinene, and Isobutylangelate dominant in this EO. These results are in agreement with those of Milia et al. (2020) [29] who tested the effect of EO from *Pistacia lentiscus* on the microorganisms responsible for periodontal diseases; they found a strong bacteriocidal activity (the MIC is between 3.13 and 12.5 µg/mL), and they reported that this activity is due to the presence of α-pinene and terpinen-4-ol in this EO with high percentages. On the other hand, *Cedrus atlantica* EO also showed an antibacterial effect against all microorganisms tested: bacteria and fungi. It seems reasonable to hypothesize that the activities are attributed to the sesquiterpenes found as major components in this oil, primarily β-Himachalene. Belkacem et al. (2021) [24] reported that EO derived from *Cedrus atlantica* cones showed no inhibition of *Staphylococcus aureus* and *Escherichia coli* strains, but it does inhibit *Bacillus cereus*; this activity is attributed to monoterpene hydrocarbons, mainly α-pinene. Additionally, our results do not agree with a previous study conducted by Derwich et al. (2010) [4] on EOs obtained from the leaves of *Cedrus atlantica* grown in Morocco, where the major component was α-pinene. This compound also exhibits potential antibacterial activities [61].

## 3. Materials and Methods

### 3.1. Chemicals and Reagents

The chemical products and solvents used in this study are from Sigma Aldrich, Morocco. All chemicals and solvents used were of analytical grade.

### 3.2. Plant Material

**Figure 7:**
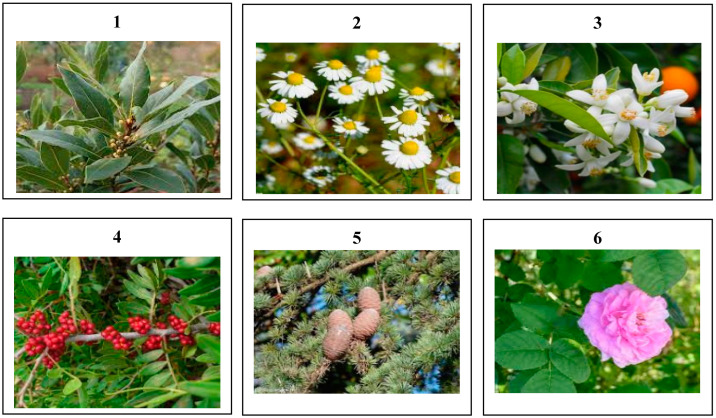
Morphological aspect of the studied plants species: **1**: *Laurus nobilis*; **2**: *Chamaemelum nobile*; **3**: *Citrus aurantium*; **4**: *Pistacia lentiscus*; **5**: *Cedrus atlantica*; and **6**: *Rosa damascena*.

The present study was carried out on samples of six species from six different regions in Morocco: *Laurus nobilis* from Meknes, *Citrus aurantium* from El Hajeb, *Chamaemelum nobile* from Tetouan, *Pistacia lentiscus* from Khenifra, *Cedrus atlantica* from Boulmane, and *Rosa damascena* from Kalaat Mggouna (Figure 7). The harvest was made between 2020 and 2021 (Table 16). After drying in the shade for two weeks, the plants were identified and authenticated at the Scientific Institute of Rabat, Botany Department.

### 3.3. Quality Control of Plant Material

#### 3.3.1. Moisture Content (MC)

The determination of the moisture content was carried out according to the AFNOR standard (NF-V03-402 1985) [62]. A quantity of 5 g of sample from each plant was weighed in previously dried and tared crucibles. The crucibles containing the vegetable matter were then placed in an oven at a temperature between 103 and 105 °C for 24 h. After this time, they were cooled in a desiccator and weighed. The moisture content was calculated using the following formula:

$$\;\mathrm{MC}\%=\left(\frac{m_{0}-m_{1}}{m_{0}}\right)\times 100$$

With:

m_0_ (g): Initial mass of the plant;

m_1_ (g): Mass after drying.

The result was expressed as a percentage of dry matter.

#### 3.3.2. pH Determination

The method entails mixing 2 g of ground sample with 10 mL of hot distilled water. The mixture was stirred then left to cool and filtered. The electrode of the pH meter was then immersed in a volume of filtrate to record the pH value.

#### 3.3.3. Determination of Titratable Acidity

Titratable acidity is the sum of free mineral and organic acids. The protocol used consists of adding 100 mL of boiling distilled water to 2 g of ground material from each plant. After stirring for 15 min, the mixture was filtered and then titrated with a solution of NaOH (N = 0.01), in the presence of a few drops of phenolphthalein, until the color changed, and a pink color was obtained which persisted for almost 30 s. The noted titration volume is converted into a citric acid equivalent by multiplying it by the factor obtained via the following calculation (method taken from Ruck (1963) and Bergeron (1995)).

$$A\%=\mathrm{Dilution}\;\mathrm{factor}\times \frac{\mathrm{weight}\;\mathrm{of}\;\mathrm{equiv},\mathrm{acid}\;\times \;\mathrm{Normality}\;\mathrm{of}\;\mathrm{NaOH}\;\times \;\mathrm{titration}\;\mathrm{volume}\;\left(\mathrm{mL}\right)}{\mathrm{Sample}\;\mathrm{weight}\;\left(g\right)}$$

#### 3.3.4. Ash Contents

The calcination of a sample of each plant serves as the foundation for the theory; 5 g of crushed sample were placed in a muffle furnace, at a temperature of 550 °C, until all carbonaceous particles were completely destroyed, and whitish ashes of constant weight were obtained [63]. The following formula was used to determine the organic matter content:

$$\;\mathrm{OM}\%=\left(\frac{W_{1}-W_{2}}{\mathrm{TS}}\right)\times 100$$

OM%: Organic matter;

W_1_: Weight of sample before calcination;

W_2_: Weight of sample after calcination;

TS: Test sample.

The ash content was calculated as follows: Ash% = 100 − MO%.

#### 3.3.5. Heavy Metal Assays: Atomic Emission Spectrometry Coupled with Induced Plasma (ICP-AES)

The standardized mineralization methodology was used to determine the levels of heavy metals in plant material, including Arsenic (As), Cadmium (Cd), Chromium (Cr), Iron (Fe), Lead (Pb), Antimony (Sb), and Titanium (Ti) [64]. The latter involves combining the crushed plant material (0.1 g) with 3 mL of aqua regia made of 1 mL of nitric acid HNO_3_ (99%) and 2 mL of hydrochloric acid HCl (37%), and then placing the mixture in a reflux assembly at 200 °C for 2 h after cooling and decantation. The supernatant was removed, filtered using a 0.45 μm membrane, and then diluted with distilled water to a volume of 15 mL. The UATRS (Technical Support Unit for Scientific Research) laboratory at CNRST in Rabat used the inductively coupled plasma atomic emission spectrophotometer ICP-AES (Ultima 2 Jobin Yvon) to measure the amounts of heavy metals.

### 3.4. Phytochemical Screening of Plant Material

Phytochemical screening was carried out on the six species studied. These tests are qualitative and based on the visual observation of hue shifts or precipitation development. In fact, the presence of reagents enables the identification of the primary and secondary metabolite families in plants. These tests for detecting groups of chemical compounds were carried out according to the protocols described in previous works [65,66].

#### 3.4.1. Primary Metabolites

The presence and nature of polysaccharides (starch, glycogen) were determined by the color of the extract (dark blue, brown) brought into contact with iodized water. However, the detection of reducing sugars was carried out by the Fehling method where the revelation of reducing sugars is indicated by the formation of a brick-red precipitate. The characterization of the proteins was carried out by two methods: the biuret reaction in which the appearance of a colored complex (mauve or violet) is obtained by adding a few drops of copper sulphate to the side in basic medium, and the xanthoprotein reaction, thanks to which the evidence of certain amino acids is revealed by heating nitric acid in contact with the solution to be analyzed [65]. The detection of lipids was carried out by adding acetic anhydride in acid medium to the extract to be analyzed; the latter turns intense red, then purple, then blue and finally takes on a dark green color. This coloration changes rapidly over time which makes it even more difficult to define specific coloration.

#### 3.4.2. Secondary Metabolites

The precipitation of salts following the use of Mayer and Dargendorff’s reagent served as evidence that alkaloids were present. When it comes to tannins, gallic tannins were characterized using the Stiasny reagent, sodium acetate, and ferric chloride, whereas concentrated hydrochloric acid and isoamyl alcohol were used to identify catechin tannins. The leuco-anthocyanins were likewise discovered by the cyanidin reaction but without the addition of magnesium chips, which were employed to highlight the free flavonoids. The addition of 10% sulfuric acid and 25% NH_4_OH allowed the detection of anthocyanins to be made. Ammonia that has been diluted by 25% was used to highlight the anthracene derivatives. With the use of the right chemicals and potassium hydroxide, cardiotonic glycosides were found. Triterpenes and sterols were revealed by the addition of strong sulfuric acid. By evaluating the foam index of each sample, saponosides—which are distinguished by their ability to froth in aqueous solution—were discovered. The aqueous decoction was given a characterizing boost by the addition of 100% ethanol. The aqueous decoction is then enhanced with strong sulfuric acid and an ethanol solution that has been soaked with thymol to bring out the oses and holosides [66].

### 3.5. Extraction and Quality Control of EOs

#### 3.5.1. Extraction and Determination of EOs Yields

The extraction of EOs from the plants studied was carried out by the hydrodistillation technique. Indeed, a quantity of 100 g of plant material, from each dried plant, was placed with 1 L of water in a flask topped with a Clevenger and a cooler. The heating mantle was set at a temperature of 90 °C. The distillation lasted 3 h after recovery of the first drop of distillate. The oils obtained were then dried by adding anhydrous sodium sulphate (Na_2_SO_4_) and stored at a temperature of 4 °C in an airtight brown glass bottle until use. The yield of EO extraction was expressed in volume of EO per mass of plant material (V/m) according to the following formula [67]:

$$\;\mathrm{Yield}\%=\left[\frac{V}{W_{0}-(W_{0}\times \mathrm{MC}\%)}\times 10^{4}\right]\mp \mathrm{SD}$$

With:

MC%: Percentage of moisture of plant material (moisture content);

W_0_: Mass of plant material distilled (g);

V: Volume of essential oil collected (ml);

SD: Standard deviation.

#### 3.5.2. Analysis and Identification of the Chemical Composition of EOs

The analysis of the EOs’ chromatography was done using a gas chromatograph of the Thermo Electron type (Trace GC Ultra) coupled to a mass spectrometer of the Thermo Electron Trace MS system type (Thermo Electron: Trace GC Ultra; Polaris Q MS), where the fragmentation is done by electron impact with a 70 eV intensity. The chromatograph has a flame ionization detector (FID) driven by an H2/Air gas mixture and a DB-5 type column (5% phenyl-methyl-siloxane) measuring 30 m × 0.25 mm × 0.25 μm film thickness. For 5 min, the column temperature will rise at a rate of 4 °C/min from 50 to 200 °C. Split injection is employed, with a leakage ratio of 1/70 and a flow rate of 1 mL/min for the vector gas nitrogen. By comparing the calculated Kovats indices (IK) of EOs with those of Adams and other reference products that were known to exist in the literature [68,69], the chemical composition of EOs was identified. It was enhanced by comparing indices and mass spectra with other references [68].

#### 3.5.3. EO Quality Control

##### Density

The density of an EO at 20 °C corresponds to the ratio of the density of this oil to the density of pure water at the same temperature. It was determined according to the following formula:

$$\;d_{20\;}=\frac{m_{2}-m_{0}}{m_{1}-m_{0}}$$

With:

$m_{0\left(g\right)\;}$: Mass of the empty pycnometer;

$m_{1\left(g\right)\;}$: Mass of the pycnometer filled with water;

$m_{2\left(g\right)}$: Mass of the pycnometer filled with oil.

##### Acid Value

It represents the proportion of free fatty acids, which appear when the triglycerides in olive oil are broken down. This rate was expressed in “grams of free oleic acid per 100 g of oil”. This value was determined according to regulation CEE/2568/91. Indeed, 0.5 g of oil was placed in a beaker with 15 mL of ethanol in the presence of a few drops of phenolphthalein used as a colored indicator. The oil was then neutralized with a solution of potassium hydroxide (Ethanolic KOH) of known titer (0.1 N). The acidity expressed in % of oleic acid and the acid value was calculated, respectively, according to the following formulas:

$$\;\mathrm{Acidity}\%=\frac{V\;\times \;N\;\times \;\mathrm{MW}}{10\;\times \;\mathrm{TS}}\;\;\;\mathrm{Acid}\;\mathrm{value}=\frac{N\;\times \left(V-V_{0}\right)\times {\;M}_{\mathrm{KOH}}}{\mathrm{TS}}$$

With:

V (ml): Volume of burette drop;

V_0_ (ml): Volume of KOH used for blank titration;

N: Normality of the KOH solution;

TS (g): Test sample;

MW: Molecular weight of oleic acid = 282 g/mol.

##### Iodine Value

The iodine value of a body is the mass of iodine in grams that can be fixed per 100 g of fatty substances; it makes it possible to evaluate the degree of establishment according to the ISO 3961 method. As the iodine binds very slowly to the double bonds, iodine chloride (Wijs’ reagent) is used; this reacts more easily. A mass of 0.1 g of the oil was placed in a 100 mL Erlenmeyer flask containing 4 mL of chloroform and 5 mL of Wijs’ reagent. The solution was gently stirred and then placed away from light for 1 h at 20 °C. Then, 4 mL of 10% KI and 30 mL of distilled water were added. The sample was titrated with sodium thiosulfate (0.1 N) in the presence of a few drops of 0.5% starch paste. A blank test without oil was carried out under the same conditions. The Iodine Index was calculated according to the following formula:

$$\;I\;V\;=\frac{126.9\;\times \;N\;\times \;\left(V_{0}-V_{1}\right)}{\mathrm{TS}}$$

With:

V_0_: Volume of sodium thiosulfate used for the blank titration;

V_1_: Volume of sodium thiosulfate solution;

N: Normality of the thiosulfate solution;

TS: Test sample.

##### Peroxide Value

The peroxide value of oil is a check on the progress of the first oxidation stage. The higher this value, the more the oil is oxidized. According to regulation CEE/2568/91, 0.5 g of the oil is weighed in a 100 mL container, then added with 4 mL of dichloromethane, 6 mL of acetic acid, and 0.1 mL of saturated aqueous potassium iodide solution (15 g of potassium iodide in 10 mL of distilled water). The flask is immediately capped and shaken for 1 min. The flask is left for 5 min in the dark. Then, added with 10 mL of distilled water. The titration is carried out by shaking vigorously and in the presence of starch paste as a colored indicator; the iodine released is measured with the 0.01 N sodium thiosulphate solution. The peroxide value (PV) is expressed in milliequivalents of oxygen per kg of oil.

$$\;\mathrm{PV}=\frac{V\;\times 1000\times \;N}{\mathrm{TS}}$$

With:

V (mL): Volume of sodium thiosulfate;

TS (g): Test sample of the oil sample to be analyzed;

N: Titer of the sodium thiosulfate solution.

##### Saponification Value

The saponification value corresponds to the mass of potash (KOH) necessary to saponify the fatty acid esters and neutralize the non-esterified fatty acids in one gram of fat/fatty substance. According to ISO 3657, the higher the molar mass, the lower the saponification value. In order to determine the saponification value, 1 g of sample with 12.5 mL of 0.5 N ethanolic potassium hydroxide solution was placed in a reflux assembly and heated at 80 °C for 1 h. Then, we added 0.5 mL of phenolphthalein to the hot solution. The cooled sample was titrated with 0.5 N HCl until the pink color completely disappeared. The blank was made under the same conditions without the oil. The results were expressed as follows:

$$\;S\;V=\frac{(V_{0\;}-V_{1})\times \;N\;\times 56.11}{\mathrm{TS}}$$

With:

V_0_: Volume of HCl in the blank test;

V_1_: Cruet drop;

N: Normality of HCl;

TS: Test sample;

M = 56.11 g/mol: Molar mass of KOH.

### 3.6. Antimicrobial Activity

#### 3.6.1. Microbial Material

The determination of the antimicrobial activity of EOs from different plants studied was carried out on sixteen microorganisms (Table 17) including eight bacterial strains and eight fungal strains. These microorganisms are pathogenic, known for their strong resistance to the antibiotics Gentamicin, Amoxicillin–Clavulanate, Vancomycin, and Trimethoprim–Sulfamethoxazole. All strains were taken from a 20% glycerol stock at −80 °C, rejuvenated on Mueller Hinton and Sabouraud broths and subcultured before use.

#### 3.6.2. Determination of Minimum Inhibitory Concentration, Minimum Bactericidal Concentration, and Minimum Fungicidal Concentration

The minimum inhibitory concentration (MIC) corresponds to the lowest concentration of the EO which produces complete inhibition of the growth of microorganisms. The MIC was determined using the microdilution method [70]. In fact, a series of dilutions were performed from a stock solution of the EO produced in DMSO at 10% to obtain concentrations of 5 to 0.93 × 10^−2^ mg/mL of each EO. For a final volume of 100 μL for each concentration, these dilutions were made in Mueller Hinton broth medium for bacteria and in Sabouraud broth for fungus. Then, 100 µL of the microbial inoculum with a final concentration of 10^6^ or 10^4^ CFU/mL in the case of bacteria or fungi, respectively, were added to the different concentrations of the dilution series. After incubation for 24 h at 37 °C, 10 µL of resazurin was added to each well as a measure of bacterial growth. The hue of the sample changed from purple to pink after a second incubation at 37 °C for 2 h. The lowest concentration that prevents resazurin from changing color is determined to be the MIC value. The 11th and 12th wells in each series were regarded as the growth and sterility controls, respectively. For each oil, the test was conducted twice more. An amount of 10 μL of each well without visible growth was obtained in order to estimate the minimum bactericidal concentration (MBC) or minimum fungicidal concentration (MFC). These samples were then inoculated into Mueller Hinton (MH) agar for bacteria or Sabouraud for fungi, respectively. At 37 °C, the plates were incubated for 24 h. The lowest sample concentration that resulted in a 99.99% reduction in CFU/mL relative to the control was used to determine MBC and MFC. The antibacterial potency was evaluated using the MBC/MIC or MFC/MIC ratio. The EO effect is bacteriocidal/fungicidal if this ratio is less than 4 and bacteriostatic/fungistatic if it is larger than 4.

### 3.7. Antioxidant Activity of EOs by the Antiradical DPPH Method

The purpose of this test is to determine whether EOs have any antiradical effects using a stable free radical called 2,2-diphenyl-1-picrylhydrazyl (DPPH). The DPPH-trapping test was performed using the procedure outlined by Liu et al. (2009) [71]. An amount of 100 mL of pure methanol was used to dissolve 2.4 mg of DPPH to create the DPPH solution. Then, 100 µL of each EO were combined with 3.9 mL of DPPH solution to conduct the test. The discoloration compared to the negative control containing only the DPPH solution was evaluated at 517 nm after the samples were placed in the dark for 30 min. Moreover, pure methanol was used to run a blank. Three times each were done for each test. Ascorbic acid is the reference material and is utilized in various amounts. In terms of a percentage reduction in DPPH (AA%), the results were as follows:

$$\;\mathrm{AA}\%=\frac{A_{\mathrm{control}}-A_{\mathrm{sample}}}{A_{\mathrm{control}}}\times 100$$

AA (%): Percentage of antioxidant activity;

A _control_: Absorbance of the solution containing only the solution of the DPPH radical;

A _sample_: Absorbance of the solution of the samples to be tested in the presence of DPPH.

The inhibitory concentration of EOs might be calculated using the graph of absorbance change as a function of ascorbic acid concentration. Results are frequently plotted against a reference antioxidant, such as ascorbic acid, because there is no exact way to evaluate a compound’s capacity for antioxidants.

### 3.8. Statistical Analysis

The data were presented as the mean ± standard mean error (SME). The statistical significance was set at *p* < 0.05. Using matrices of the Pearson type, principal component analysis (PCA) was carried out. Using dissimilarity matrices estimated in Euclidean distance, hierarchical cluster analyses (HCA) and the dendrogram were completed, and the average link method of systematic aggregation was selected. R was used to carry out these analyses.

## 4. Conclusions

This work was devoted to the study of the chemical composition of the EO of six species, namely, *Laurus nobilis*, *Chamaemelum nobile*, *Citrus aurantium*, *Pistacia lentiscus*, *Cedrus atlantica*, and *Rosa damascena*. Phytochemical screening of these species has revealed various primary and secondary metabolites. The EOs of these plants vary according to the species. These data enabled us to confirm an intraspecific chemical polymorphism in the species studied and to show that these EOs are rich in terpene products. The presence of these compounds gives EOs very interesting antioxidant and antimicrobial properties. The EOs studied also have significant bacteriocidal activities against bacteria (Gram-negative and Gram-positive) and act as fungicides against strains of yeasts and molds. So, these oils have considerable interest as natural antimicrobial agents. These properties also give the oils of these plants therapeutic and cosmetic virtues worthy of interest; this explains the tendency of the organic industries to substitute synthetic products which are harmful to health with EOs extracted from plants.

## Figures and Tables

**Table 1:** Quality control of plant matter: moisture content (MC), pH, acidity, organic material (OM), and mineral matter (MM).

Species	MC (%)	pH	Acidity (%)	OM (%)	MM (%)
*Laurus nobilis*	14.88 ± 0.11	5.76 ± 0.01	0.85 ± 0.02	96.33	3.67
*Chamaemelum nobile*	18.01 ± 0.27	4.55 ± 0.02	1.73 ± 0.06	91.95	8.05
*Citrus aurantium*	83.61 ± 0.20	4.78 ± 0.01	3.04 ± 0.16	94.10	5.90
*Pistacia lentiscus*	10.27 ± 0.22	4.03 ± 0.02	0.18 ± 0.02	81.77	18.23
*Cedrus atlantica*	13.33 ± 0.31	5.41 ± 0.01	0.21 ± 0.10	71.86	28.14
*Rosa damascena*	10.29 ± 0.29	4.93 ± 0.01	4.19 ± 0.06	95.02	4.98

**Table 2:** Concentration of heavy metals (mg/L) (ICP) and FAO/WHO Maximum Limit (2009).

Species	Chrome(Cr)	Antimony (Sb)	Lead (Pb)	Cadmium (Cd)	Iron (Fe)	Titanium (Ti)
*Laurus nobilis*	0.0003	0.0126	Undetectable	Undetectable	0.4512	0.0102
*Chamaemelum nobile*	0.0011	0.0102	Undetectable	Undetectable	1.166	0.0135
*Citrus aurantium*	0.0002	0.008	Undetectable	Undetectable	0.3816	0.0043
*Pistacia lentiscus*	Undetectable	0.0131	0.0018	Undetectable	1.002	0.0083
*Cedrus atlantica*	ND	ND	ND	ND	ND	ND
*Rosa damascena*	Undetectable	0.0081	Undetectable	Undetectable	0.4369	0.0056
Maximum Limit	2	1	3	0.3	20	–

ND: not determined.

**Table 3:** Screening of the primary metabolites of the plants studied.

Species	Lipids	Proteins		Reducing Sugars	Polysaccharides
		Rx of Biuret	Rxxanthoproteic		
*Laurus nobilis*	+++	++	+++	++	+++
*Chamaemelum nobile*	++	++	++	+	++
*Citrus aurantium*	+	+++	+++	++	+
*Pistacia lentiscus*	+++	+++	+++	++	−
*Cedrus atlantica*	−	+	++	+	−
*Rosa damascena*	−	+	++	−	+

(−): Absence of the desired compound in the reaction. (+): Low presence of the tested compound. (++): Presence of the compound, with relatively high concentration. (+++): Presence of the compound with high concentration.

**Table 4:** Screening secondary metabolites of the plants studied.

Species	Tannins			Flavonoids			Alkaloids		Mucilage	Saponosides
	Totals	Catechists	Gallic	Flavonoids	Cyanidin Reaction	Anthocyanes	Dragendorff	Mayer		
*Laurus nobilis*	++	+++	+++	++	+	−	−	−	++	+
*Chamaemelum nobile*	++	++	−	++	−	−	−	−	−	−
*Citrus aurantium*	++	−	++	++	−	−	−	−	+++	−
*Pistacia lentiscus*	+++	+++	+++	+	−	+	−	−	++	+
*Cedrus atlantica*	+	+	−	+	−	−	−	−	−	−
*Rosa damascena*	+++	+++	+++	+++	++	+	−	−	+++	+

(−): Absence of the desired compound in the reaction. (+): Low presence of the tested compound. (++): Presence of the compound, with relatively high concentration. (+++): Presence of the compound with high concentration.

**Table 5:** The chemical composition of EO of *Laurus nobilis*.

RA (%)	IK	Compounds
0.63	930	Thujene<α->
5.29	939	Pinene<α->
0.15	954	Camphene
12.08	975	Sabinene
4.06	979	Pinene<β->
0.72	990	Myrcene
0.17	1002	Phellandrene<α->
1.85	1029	Limonene
1.07	1029	Phellandrene<β->
36.58	1031	Cineole<1,8->
0.68	1059	Terpinene<γ->
0.56	1070	Sabinene hydrate <*cis*->
0.69	1088	Terpinolene
3.45	1096	Linalool
0.33	1098	Sabinene hydrate <*trans*->
0.39	1166	Terpineol<δ->
1.49	1177	Terpinen-4-ol
3.15	1188	Terpineol<α->
0.16	1285	Bornylacetate
0.59	1317	Terpinylacetate<δ->
15.42	1349	Terpinylacetate<α->
1.57	1359	Eugenol
0.5	1390	Elemene<β->
5.34	1403	Methyleugenol
0.15	1500	Bicyclogermacrene
0.15	1513	Cadinene<γ->
0.46	1523	Cadinene<δ->
0.26	1541	Copaen-11-ol <α->
0.41	1557	Elemicin
0.71	1578	Spathulenol
0.18	1592	Viridiflorol
0.16	1602	Ledol
0.41	1654	Cadinol<α->
0.17	1689	Shyobunol
Compounds identified (%)		99.98
Oxygenated monoterpenes (%)		47.52
Monoterpenes (%)		27.39
Oxygenated sesquiterpenes (%)		23.81
Sesquiterpenes (%)		1.26

RA: Relative abundance (%); IK: Index Kovats.

**Table 6:** The chemical composition of EO of C*hamaemelum nobile*.

RA (%)	IK	Compounds
0.26	911	Isobutylisobutyrate
0.27	912	Tiglicacid
1.01	960	2-Methylallyl isobutyrate
0.25	1001	Isobutyl 2-methylbutanoate
0.27	1014	2-Methylbutyl isobutyrate
0.79	1020	2-Methylallyl 2-methylbutanoate
1.46	1051	Isobutylangelate
1.36	1060	2-Methylbut-2-en-1-yl isobutyrate
19.39	1112	Methallylangelate
0.69	1147	Isoamylangelate
1.03	1158	2-Methylbutyl angelate
0.20	1160	Valeric acid, 3-methylbut-2-enyl ester
0.52	1190	Prenylangelate
0.78	1193	(*Z*)-(*E*)-2-Methylbut-2-en-1-yl 2-methylbut-2-enoate
41.79	1200	AngelylAngelate
0.22	1230	2-Butenoic acid, 3-methyl-, 3-methyl-2-butenyl ester
0.11	1237	Pulegone
19.33	1243	2-hydroxy-2-methyl-3-butenyl Angelate
0.23	1252	Methylpentylangelate<3->
0.24	1290	Thymol
2.15	1299	Carvacrol
1.08	1300	2-hydroxy-2-methyl-3-butenyl Angelate
0.12	1317	Hexenyltiglate<(3*E*)->
1.83	1321	Hexenyltiglate<(3*Z*)->
1.12	1366	Decanoicacid
0.20	1480	Curcumene<ar->
0.18	1497	Benzyltiglate
0.15	1583	Caryophylleneoxide
0.16	1640	Cadinol<epi-α->
0.56	1800	Psoralen, 3-(α,α-dimethylallyl)-
0.94	1840	Psoralen, 3-(α,α-dimethylallyl)-
0.18	1900	Corymbolone
0.69	1940	1-Phenyldodec-1-en-3-one
Compounds identified (%)		99.56
Oxygenated monoterpenes (%)		94.32
Oxygenated sesquiterpenes (%)		5.04
Sesquiterpenes (%)		0.20
Monoterpenes (%)		0.00

RA: Relative abundance (%); IK: Index Kovats.

**Table 7:** The chemical composition of EO of *Citrus aurantium*.

RA (%)	IK	Compounds
0.18	939	Pinene<α->
0.57	975	Sabinene
3.98	979	Pinene<β->
1.06	990	Myrcene
6.42	1029	Limonene
0.67	1037	Ocimene<(*Z*)-β->
0.07	1042	Benzeneacetaldehyde
5.93	1050	Ocimene<(*E*)-β->
0.05	1059	Terpinene<γ->
0.10	1072	Linalooloxide<*cis*->
0.44	1088	Terpinolene
29.01	1096	Linalool
0.11	1177	Terpinen-4-ol
5.47	1188	Terpineol<α->
1.05	1229	Nerol
3.09	1252	Geraniol
12.46	1257	Linaloolacetate
0.22	1291	Indole
0.25	1337	Anthranilate<methyl->
0.17	1349	Terpinylacetate<α->
2.42	1361	Nerylacetate
4.57	1381	Geranylacetate
0.36	1419	Caryophyllene<(*E*)->
0.19	1500	Bicyclogermacrene
0.06	1505	Farnesene<(*E*,*E*)-α->
6.91	1563	Nerolidol<(*E*)->
0.2	1583	Nerylisovalerate
0.1	1689	Farnesol<2,3-dihydro->
0.29	1700	Heptadecane<n->
12.77	1743	Farnesol<(2*E*,6*E*)->
0.09	1846	Farnesylacetate<(2*E*,6*E*)->
0.07	1878	Cubitene
0.08	1900	Nonadecane<n->
0.4	1938	Cembrene
Compounds identified (%)		99.81
Oxygenated sesquiterpenes (%)		39.69
Oxygenated monoterpenes (%)		39.15
Monoterpenes (%)		19.52
Sesquiterpenes (%)		1.45

RA: Relative abundance (%); IK: Index Kovats.

**Table 8:** The chemical composition of EO of *Pistacia lentiscus*.

RA (%)	IK	Compounds
1.63	911	Isobutylisobutyrate
10.16	939	Pinene<α->
0.44	950	2-Methylallyl isobutyrate
0.88	954	Camphène
1.27	979	Pinene<β->
0.51	990	Myrcene
0.92	995	Isobutyl-(2*E*)-butenoate
0.55	1025	Allyltiglate
0.66	1038	Propyltiglate
10.65	1051	Isobutylangelate
0.42	1055	Pentylisobutanoate
3.46	1088	Terpinolene
1.22	1088	Butylangelate
1.62	1090	3-Methylbutylmethacrylate
1.74	1091	Pentylbutanoate
0.99	1093	Isobutyl 2-methylbutanoate
1.33	1096	Linalool
1.22	1100	Methyl butyl-2-methyl butyrate <2->
8.4	1112	Methallylangelate
1.62	1151	Hexylisobutanoat
2.99	1164	Pinocarvone
1.33	1177	Terpinen-4-ol
2.10	1185	n-Hexylmethacrylate
5.79	1190	pentyl 2-methylisocrotonate
2.84	1190	Prenylangelate
1.45	1195	Myrtenal
0.88	1197	Butanoic acid, 2-methyl-4-methylpentyl ester
0.66	1243	2-hydroxy-2-methyl-3-butenyl Angelate
27.83	1252	Methylpentylangelate<3>
0.72	1419	Caryophyllene<(*E*)->
3.29	1481	Germacrene D
0.43	1505	Farnesene<(*E*,*E*)-α->
Compounds identified (%)		100
Oxygenated monoterpenes (%)		49.35
Oxygenated sesquiterpenes (%)		28.71
Monoterpenes (%)		17.50
Sesquiterpenes (%)		4.44

RA: Relative abundance (%); IK: Index Kovats.

**Table 9:** The chemical composition of EO of *Cedrus atlantica*.

RA (%)	IK	Compounds
0.46	1026	Menthene<1-ρ->
0.34	1319	Cycloisolongifolene<didehydro->
0.20	1352	Longipinene<α->
0.76	1371	Cyclosativene
0.56	1407	Longifolene
1.75	1446	Vestitenone
15.74	1451	Himachalene<α->
0.68	1477	Chamigrene<β->
10.49	1482	Himachalene<γ->
2.11	1495	Cadina-1,4-diene <*cis*->
40.19	1500	Himachalene<β->
0.30	1503	Dihydroagarofuran<β->
1.39	1517	Himachalene<α-dehydro-ar->
2.16	1523	Cadinene<δ->
0.22	1529	Calamenene<cis->
2.92	1532	Himachalene<γ-dehydro-ar->
1.24	1545	Calacorene<α->
1.87	1555	Vetivenene<β->
0.29	1565	Calacorene<β->
0.83	1579	Himachaleneepoxide
0.40	1599	Longiborneol
0.94	1616	Himachaleneoxide<β->
1.14	1619	Cubenol<1,10-di-epi->
0.61	1626	Isolongifolanone<*trans*->
0.43	1653	Himachalol
1.60	1662	Allohimachalol
1.89	1685	Bisabolol<α->
1	1694	Atlantone<(*Z*)-γ->
2.22	1706	Atlantone<(*E*)-γ->
4.57	1709	Thujopsenal<*cis*->
0.67	1718	Atlantone<(*Z*)-α->
Compounds identified (%)		99.97
Sesquiterpenes (%)		81.16
Oxygenated sesquiterpenes (%)		18.35
Monoterpenes (%)		0.46
Oxygenated monoterpenes (%)		0

RA: Relative abundance (%); IK: Index Kovats.

**Table 10:** The chemical composition of EO of *Rosa damascene*.

RA (%)	IK	Compounds
0.63	1038	2-Propanol, 1,1′-oxybis-
0.8	1100	Dipropylene glycol
0.28	1180	3,5-Dihydroxy-6-methyl-2,3-dihydro-4*H*-pyran-4one
0.17	1403	Methyleugenol
0.52	1566	Dodecanoicacid
0.63	1585	8-Heptadecene
0.37	1612	Tetradecanal
5.35	1700	Heptadecane<n->
0.37	1723	Methylmyristate
0.47	1800	Octadecane<n->
12.98	1860	1-Nonadecene
44.89	1900	Nonadecane<n->
0.34	1960	Hexadecanoicacid
3.52	2000	Eicosane<n->
19.49	2100	Heneicosane<n->
1.57	2200	17-Pentatriacontene
0.44	2250	Henicos-1-ene
0.34	2300	Phenethyltetradecanoate
0.3	2320	Anthracene, 9-propyl-
4.04	2400	Tetracosane<n->
0.43	2420	Citronellyl benzoate
0.34	2450	5-Eicosene, (*E*)-
0.33	2460	2-Phenylethyl nonanoate
0.28	2460	Oleicacid,3-(octadecyloxy)propyl ester
Compounds identified (%)		98.88
Sesquiterpenes (%)		94.02
Oxygenated sesquiterpenes (%)		3.15
Oxygenated monoterpenes (%)		1.71
Monoterpenes (%)		0.00

RA: Relative abundance (%); IK: Index Kovats.

**Table 11:** Percentage of the chemical family composition of EOs of plants species.

Species	Percentage (%)								
	Ethers	Alkenes	Esters	Alcohols	Acids	Ketones	Hydrocarbons	Amines	Aldehydes
*Laurus nobilis*	42.33	28.65	16.17	12.83	0.00	0.00	0.00	0.00	0.00
*Chamaemelum nobile*	0.15	0.00	94.29	2.55	1.39	0.98	0.2	0.00	0.00
*Citrus aurantium*	0.00	0.00	20.16	58.61	0.00	0.00	20.75	0.22	0.07
*Pistacia lentiscus*	0.00	0.00	72.18	2.66	0.00	2.99	20.72	0.00	1.45
*Cedrus atlantica*	2.07	0.00	0.00	5.46	0.00	19.81	68.06	0.00	4.57
*Rosa damascena*	0.17	0.00	1.75	1.43	0.86	0.28	94.02	0.00	0.37

**Table 12:** Quality control of the EOs of plants species.

EO	Density (g/mL)	Acidity	Iodine	Peroxide	Saponification
*Laurus nobilis*	0.912 ± 0.001	0.5 ± 0.03	0.812 ± 0.01	4 ± 0.08	0.471 ± 0.04
*Chamaemelum nobile*	0.921 ± 0.002	2.24 ± 0.01	0.989 ± 0.01	30 ± 0.2	0.418 ± 0.02
*Citrus aurantium*	0.873 ± 0.001	0.56 ± 0.03	1.1 ± 0.01	40 ± 0.5	0.594 ± 0.01
*Pistacia lentiscus*	0.878 ± 0.001	1.12 ± 0.02	0.888 ± 0.01	64 ± 0.7	0.603 ± 0.07
*Cedrus atlantica*	0.937 ± 0.001	1.68 ± 0.01	0.939 ± 0.01	170 ± 0.9	0.482 ± 0.03
*Rosa damascena*	0.873 ± 0.002	ND	ND	ND	ND

**Table 13:** MIC and MBC values (mg/mL) of the EOs studied.

Bacterial Strains	Essential Oils									
	*Laurus nobilis*		*Chamaemelum nobile*		*Citrus aurantium*		*Pistacia lentiscus*		*Cedrus atlantica*	
	MIC	MBC	MIC	MBC	MIC	MBC	MIC	MBC	MIC	MBC
*Staphylococcus epidermidis*	5	5	5	5	5	5	5	5	5	5
*Staphylococcus aureus* BLACT	5	5	5	5	5	5	5	5	5	5
*Escherichia coli*	5	5	5	5	5	5	5	5	5	5
*Escherichia coli* BLSE	5	5	5	5	5	5	5	5	5	5
*Enterobacter cloacae*	5	5	5	5	5	5	5	5	5	5
*Klebsiella pneumoniae*	5	5	5	5	5	5	5	5	5	5
*Proteus mirabilis*	5	5	5	5	5	5	5	5	5	5
*Pseudomonas aeruginosa*	5	5	5	5	5	5	5	5	5	5

**Table 14:** MIC (µg/mL) of antibiotics evaluated by BD Phoenix for selected species.

Microorganism	CMI (µg/mL) Identification Instrument and Antibiogram BD Phoenix™			
	Gentamicin	Amoxicillin–Clavulanate	Vancomycin	Trimethoprim–Sulfamethoxazole
*Staphylococcus epidermidis*	2		>8	>4/76
*Staphylococcus aureus* BLACT	<0.5		2	<10
*Escherichia coli*	2	8/2		<=1/19
*Escherichia coli* BLSE	2	>8/2		>4/76
*Enterobacter cloacae*	>4	>8/2		>4/76
*Klebsiella pneumoniae*	<=1	<=2/2		<=1/19
*Proteus mirabilis*	2	<=2/2		>1/19
*Pseudomonas aeruginosa*	2	>8/2		4/76

**Table 15:** MIC and MFC (mg/mL) of the EOs studied.

Fungal Strains	Essential oils										Terbinafine (µg/mL)
	*Laurus nobilis*		*Chamaemelum nobile*		*Citrus aurantium*		*Pistacia lentiscus*		*Cedrus atlantica*		
	MIC	MFC	MIC	MFC	MIC	MFC	MIC	MFC	MIC	MFC	CMI and CMB
*Candida albicans*	5	5	5	5	5	5	5	5	5	5	12,500
*Candida dubliniensis*	5	5	5	5	5	5	2.5	2.5	5	5	3125
*Saccharomyces cerevisiae*	5	5	5	5	5	5	2.5	5	5	5	3125
*Aspergillus niger*	5	5	5	5	5	5	5	5	5	5	3125
*Candida tropicalis*	5	5	5	5	5	5	5	5	5	5	12,500
*Candida krusei*	5	5	5	5	5	5	5	5	5	5	50,000
*Candida kyfer*	5	5	5	5	5	5	5	5	5	5	25,000
*Candida parapsilosis*	1.2	2.5	1.2	2.5	1.2	1.2	0.6	1.2	0.6	1.2	6250

**Table 16:** Distribution of individuals in the plant species studied and their harvest sites by region.

N°	Latin Name	Harvest Site		Parts Used	Latitude (x)	Longitude (y)	Altitude (m)	Harvest Year
		Region	Locality					
1	*Laurus nobilis*	Meknes	Wislane	Leaves	5.48′49″ W	33°92′52″ N	558	2020
2	*Chamaemelum nobile*	Tetouan	Beni Imrane	Leaves	5.40′09″ W	35°52′24″ N	395	2020
3	*Citrus aurantium*	El Hajeb	SbaaaAyoun	Leaves	5.37′53″ W	33°9067631 N	589	2020
4	*Pistacia lentiscus*	Khenifra	M’rirt	Leaves	5.56′92″ W	33°15′78″ N	1113	2021
5	*Cedrus atlantica*	Boulmane	Enjil	Wood	4.55′87″ W	33°18′88″ N	1639	2021
6	*Rosa damascena*	Kalaat M’Gouna	Kalaat M’Gouna	Leaves	6.12′76″ W	31°21′11″ N	1450	2020

**Table 17:** List of bacterial strains tested with their references.

		Strains	References			Strains	References
Bacteria	Gram- positive Cocci	*Staphylococcus epidermidis*	5994	Fungal	Yeasts	*Candida albicans*	Ca
		*Staphylococcus aureus* BLACT	4IH2510			*Candida dubliniensis*	Cd
	Gram-negative Bacilli	*Escherichia coli*	3DT1938			*Candida kyfer*	Cky
		*Escherichia coli* BLSE	2DT2057			*Candida parapsilosis*	Cpa
		*Enterobacter cloacae*	02EV317			*Candida tropicalis*	Ct
		*Klebsiella pneumoniae*	3DT1823			*Candida krusei*	Ckr
		*Proteus mirabilis*	2DS5461			*Saccharomyces cerevisiae*	Sacc
		*Pseudomonas aeruginosa*	2DT2138		Molds	*Aspergillus niger*	AspN

## Data Availability

Not applicable.

## Supplementary Materials

The following supporting information can be downloaded at https://www.mdpi.com/article/10.3390/antibiotics12040721/s1, Figure S1: Representative GC–MS chromatogram of EOs of plants species.

## References

[1] Newman D.J., Cragg G.M. (2020). Natural products as sources of new drugs over the nearly four decades from 01/1981 to 09/2019. J. Nat. Prod..

[2] Al-Rimawi F., Jaradat N., Qneibi M., Hawash M., Emwas N. (2020). Free radicals and enzymes inhibitory potentials of the traditional medicinal plant *Echium angustifolium*. Eur. J. Integr. Med..

[3] Sun J., Sun P., Kang C., Zhang L., Guo L., Kou Y. (2022). Chemical composition and biological activities of essential oils from six lamiaceae folk medicinal plants. Front. Plant Sci..

[4] Derwich E., Benziane Z., Boukir A. (2010). Chemical Composition and In Vitro Antibacterial Activity of the Essential Oil of *Cedrus atlantica*. Int. J. Agric. Biol..

[5] Abdoul-Latif M.F., Ainane A., Oumaskour K., Boujaber N., Jalludin M., Ainane T. (2021). Chemical Composition and Antimicrobial Activity of the Essential Oil of *Chamaemelum Nobile* (L.) All. PhOL.

[6] Ma L., Li J. (2021). Food Flavor Substances. Essentials of Food Chemistry.

[7] Czernicka M., Chłosta I., Kęska K., Kozieradzka-Kiszkurno M., Abdullah M., Popielarska-Konieczna M. (2021). Protuberances are organized distinct regions of long-term callus: Histological and transcriptomic analyses in kiwifruit. Plant Cell Rep..

[8] Sarkic A., Stappen I. (2018). Essential Oils and Their Single Compounds in Cosmetics—A Critical Review. Cosmetics.

[9] Guzmán E., Lucia A. (2021). Essential Oils and Their Individual Components in Cosmetic Products. Cosmetics.

[10] De Groot A.C., Schmidt E. (2016). Essential Oils: Contact Allergy and Chemical Composition.

[11] Drioiche A., Benhlima N., Kchibale A., Boutahiri S., Ailli A., El Hilali F., Moukaid B., Zair T. (2021). Ethnobotanical investigation of herbal food additives of Morocco used as natural dyes. Ethnobot. Res. Appl..

[12] Caputo L., Nazzaro F., Souza L.F., Luigi A., Martino L.D., Fratianni F., Coppola R., Feo V.D. (2017). *Laurus nobilis*: Composition of Essential Oil and its Biological Activities. Molecules.

[13] Dobroslavi E., Repaji M., Dragović-Uzelac V., Garofuli I.E. (2022). Isolation of *Laurus nobilis* Leaf Polyphenols: A Review on Current Techniques and Future Perspectives. Foods.

[14] Farkas P., Hollá M., Vaverková S., Stahlová B., Tekel J., Havránek E. (2003). Composition of the Essential Oil from the Flowerheads of *Chamaemelum nobile* (L.) All. (*Asteraceae*) Cultivated in Slovak Republic. J. Essent. Oil Res..

[15] Kacániová M., Terentjeva M., Galovičová L., Ivanišová E., Štefániková J., Valková V., Borotová P., Kowalczewski P.Ł., Kunová S., Felšöciová S. (2020). Biological Activity and Antibiofilm Molecular Profile of *Citrus aurantium* Essential Oil and its Application in a Food Model. Molecules.

[16] Moraes T.M., Kushima H., Moleiro F.C., Santos R.C., Machado Rocha L.R., Marques M.O., Vilegas W., Hiruma-Lima C.A. (2009). Effects of limonene and essential oil from *Citrus aurantium* on gastric mucosa: Role of prostaglandins and gastric mucus secretion. Chem. Biol. Interact..

[17] Leja K., Szudera-Kończal K., Switała E., Juzwa W., Kowalczewski P.Ł., Czaczyk K. (2019). The Influence of Selected Plant Essential Oils on Morphological and Physiological Characteristics in *Pseudomonas Orientalis*. Foods.

[18] Ouedrhiri W., Bouhdid S., Balouiri M., Lalami A.E.O., Moja S., Chahdi F.O., Greche H. (2015). Chemical composition of *Citrus aurantium* L. leaves and zest essential oils, their antioxidant, antibacterial single and combined effects. J. Chem. Pharm. Res..

[19] Amorim J.L., Simas D.L.R., Pinheiro M.M.G., Moreno D.S.A., Alviano C.S., da Silva A.J.R., Dias Fernandes P. (2016). Anti-Inflammatory Properties and Chemical Characterization of the Essential Oils of Four Citrus Species. PLoS ONE.

[20] Khakpour S., Khosravi M., Mashayekhipour Z., Jahromy M.H. (2014). Effect of *Citrus aurantium* L. Essential Oil and Haloperidol on Anxiety in Male Mice. World J. Neurosci..

[21] Tabanca N., Demirci B., Nalbantsoy A., Kendra P.E., Demirci F., Demirci B. (2020). Chemical Characterization and Biological Activity of the Mastic Gum Essential Oils of *Pistacia lentiscus* var. Chia from Turkey. Molecules.

[22] Xynos N., Termentzi A., Fokialakis N., Skaltsounis L.A., Aligiannis N. (2018). Supercritical CO_2_ extraction of mastic gum and chemical characterization of bioactive fractions using LC-HRMS/MS and GC–MS. J. Supercrit. Fluids.

[23] Paoli M., Nam A.M., Castola V., Casanova J., Bighelli A. (2011). Chemical Variability of the Wood Essential Oil of *Cedrus atlantica* Manetti from Corsica. Chem. Biodivers..

[24] Belkacem N., Khettal B., Hudaib M., Bustanji Y., Abu-Irmaileh B., Amrine C.S.M. (2021). Antioxidant, antibacterial, and cytotoxic activities of *Cedrus atlantica* organic extracts and essential oil. Eur. J. Integr. Med..

[25] Nunes H.S., Miguel M.G. (2017). *Rosa damascena* essential oils: A brief review about chemical composition and biological properties. Trends Phytochem. Res..

[26] Harborne A.J. (1998). Phytochemical Methods a Guide to Modern Techniques of Plant Analysis.

[27] Bruneton J. (1999). Pharmacognosie, Phytochimie, Plantes Médicinales.

[28] Paun G., Zrira S., Boutakiout A., Ungureanu O., Simion D., Chelaru C., Radu G.L. (2013). Chemical composition, antioxidant and antibacterial activity of essential oils from moroccan aromatic herbs. Rev. Roum. Chim..

[29] Milia E., Usai M., Szotáková B., Elstnerová M., Králová V., D’hallewin G., Spissu Y., Barberis A., Marchetti M., Bortone A. (2020). The Pharmaceutical Ability of *Pistacia lentiscus* L. Leaves Essential Oil Against Periodontal Bacteria and *Candida sp*. and Its Anti-Inflammatory Potential. Antibiotics.

[30] El Idrissi M., Idrissi E.L. (2014). Caractérisation Chimique de Certaines Espèces de Thym Marocain Du Moyen Atlas (Région de Khenifra). Sci. Lib..

[31] Fidan H., Stefanova G., Kostova I., Stankov S., Stoyanova A., Zheljazkov V.D. (2019). Chemical Composition and Antimicrobial Activity of *Laurus nobilis* L. Essential Oils from Bulgaria. Molecules.

[32] Ekren S., Yerlikaya O., Tokul H.E., Akpınar A., Acu M. (2013). Chemical composition, antimicrobial activity and antioxidant capacity of some medicinal and aromatic plant extracts. Afr. J. Microbiol. Res..

[33] Snuossi M., Trabelsi N., Ben Taleb S., Dehmeni A., Flamini G., de Feo V. (2016). *Laurus nobilis*, *Zingiber officinale* and *Anethum graveolens* essential oils: Composition, antioxidant and antibacterial activities against bacteria isolated from fish and shellfish. Molecules.

[34] Derwich E., Benziane Z., Boukir A. (2009). Chemical composition and antibacterial activity of leaves essential oil of *Laurus nobilis* from Morocco. Aust. J. Basic Appl. Sci..

[35] Yalcin H., Anik M., Sanda M.A., Cakir A. (2007). Gas chromatography/mass spectrometry analysis of *Laurus nobilis* essential oil composition of northern Cyprus. J. Med. Food..

[36] Roozbeh F., Dong-Jin L. (2017). P 021—Chemical constituents and antioxidant properties of *Matricaria recutita* and *Chamaemelum nobile* essential oil growing in south west of Iran. Free Radic. Biol. Med..

[37] Aremu O.O., Tata C.M., Sewani-Rusike C.R., Oyedeji A.O., Oyedeji O.O., Nkeh-Chungag B.N. (2018). Phytochemical composition, and analgesic and anti-inflammatory properties of essential oil of *Chamaemelum nobile* (Asteraceae L All) in rodents. Trop. J. Pharm. Res..

[38] Bourgou S., Rahali F.Z., Ourghemmi I., Saïdani Tounsi M. (2012). Changes of Peel Essential Oil Composition of Four Tunisian Citrus during Fruit Maturation. Sci. World J..

[39] Sarrou E., Chatzopoulou P., Dimassi-Theriou K., Therios I. (2013). Volatile constituents and antioxidant activity of peel, flowers and leaf oils of *Citrus aurantium* L. growing in Greece. Molecules.

[40] Duru M.E., Cakir A., Kordali S., Zengin H., Harmandar M., Izumi S., Hirata T. (2003). Chemical composition and antifungal properties of essential oils of three Pistacia species. Fitoterapia.

[41] Aberchane M., Fechtal M., Chaouch A. (2004). Analysis of Moroccan Atlas Cedarwood Oil (*Cedrus atlantica* Manetti). J. Essent. Oil Res..

[42] Boudarene L., Baaliouamer A., Meklati B.Y., Scharff C. (2004). Composition of the Seed Oils from Algerian *Cedrus atlantica* G. Manetti. J. Essent. Oil Res..

[43] Ghavam M., Afzali A., Manconi M., Bacchetta G., Manca M.L. (2021). Variability in chemical composition and antimicrobial activity of essential oil of *Rosa × damascena* Herrm. from mountainous regions of Iran. Chem. Biol. Technol. Agric..

[44] Angioni A., Barra A., Coroneo V., Dessi S., Cabras P. (2006). Chemical composition, seasonal variability, and antifungal activity of *Lavandula stoechas* L. ssp. stoechas essential oils from stem/leaves and flowers. J. Agric. Food. Chem..

[45] Ordoudi S.A., Papapostolou M., Nenadis N., Tsimidou M.Z., Mantzouridou F.T. (2022). Bay Laurel (*Laurus nobilis* L.) Essential Oil as a Food Preservative Source: Chemistry, Quality Control, Activity Assessment, and Applications to Olive Industry Products. Foods.

[46] Zarith Asyikin A.A., Akil A., Siti Hamidah M.S., Alptug K., Muhammad Mohsin A., David L., Mohd R., Magdah G., Mohammad A.K., Ghulam M.A. (2018). Essential Oils: Extraction Techniques, Pharmaceutical and Therapeutic Potential—A Review. Curr. Drug Metab..

[47] Shanaida M., Hudz N., Białon M., Kryvtsowa M., Svydenko L., Filipska A., Wieczorek P.P. (2021). Chromatographic profiles and antimicrobial activity of the essential oils obtained from some species and cultivars of the Mentheae tribe (Lamiaceae). Saudi J. Biol. Sci..

[48] González-Mas M.C., Rambla J.L., López-Gresa M.P., Blázquez M.A., Granell A. (2019). Volatile Compounds in Citrus Essential Oils: A Comprehensive Review. Front. Plant Sci..

[49] Fortes G.A.C., Naves S.S., Godoi F.F.F., Duarte A.R., Ferri P.H., Santos S.C. (2011). Assessment of a Maturity Index in Jabuticaba Fruit by the Evaluation of Phenolic Compounds, Essential Oil Components, Sugar Content and Total Acidity. Am. J. Food Technol..

[50] Farahmandfar R., Asnaashari M., Pourshayegan M., Maghsoudi S., Moniri H. (2018). Evaluation of antioxidant properties of lemon verbena (*Lippia citriodora*) essential oil and its capacity in sunflower oil stabilization during storage time. Food Sci. Nutr..

[51] (2009). Codex Alimentarius. International Food Standards for Named Vegetable Oils. Adopted in 1999. Amendment: 2005, 2011, 2013, 2015. Revision: 2001, 2003, 2009.

[52] Asnaashari E., Asnaashari M., Ehtiati A., Farahmandfar R. (2015). Comparison of adaptive neuro-fuzzy inference system and artificial neural networks (MLP and RBF) for estimation of oxidation parameters of soybean oil added with curcumin. J. Food Meas. Charact..

[53] Ibipiriene E.-F., Akpa J.G., Ehirim E.O. (2022). Comparative Study on the Analysis and Utilization of Citrus Peels Essential Oil and Pectin. Iconic Res. Eng. J..

[54] Guenane H., Gherib A., Carbonell-Barrachina Á., Cano-Lamadrid M., Krika F., Berrabah M., Maatallah M., Bakchiche B. (2016). Minerals analysis, antioxidant and chemical composition of extracts of *Laurus nobilis* from southern Algeria. J. Mater. Environ. Sci..

[55] Mssillou I., Agour A., El Ghouizi A., Hamamouch N., Lyoussi B., Derwich E. (2020). Chemical Composition, Antioxidant Activity, and Antifungal Effects of Essential Oil from *Laurus nobilis* L. Flowers Growing in Morocco. J. F. Qual..

[56] Ben Hsouna A., Hamdi N., Ben Halima N., Abdelkafi S. (2013). Characterization of Essential Oil from *Citrus aurantium* L. Flowers: Antimicrobial and Antioxidant Activities. J. Oleo Sci..

[57] Wang Y., Li X., Jiang Q., Sun H., Jiang J., Chen S., Guan Z., Fang W., Chen F. (2018). GC-MS Analysis of the Volatile Constituents in the Leaves of 14 Compositae Plants. Molecules.

[58] Baser K.H.C., Buchbauer G. (2015). Handbook of Essential Oils: Science, Technology, and Applications.

[59] Ansari Dezfooli N., Hasanzadeh N., Bagher Rezaee M., Ghasemi A. (2012). Antibacterial Activity and Chemical Compositions of *Chamaemelum nobile* Essential Oil/Extracts against *Psedomonas tolaasii*, the Causative Agent of Mushroom Brown Blotch. Anna. Biol. Res..

[60] Ben Hsouna A., Ben Halima N., Smaoui S., Hamdi N. (2017). *Citrus lemon* essential oil: Chemical composition, antioxidant and antimicrobial activities with its preservative effect against *Listeria monocytogenes* inoculated in minced beefmeat. Lipids Health Dis..

[61] Saab A.M., Gambari R., Sacchetti G., Guerrini A., Lampronti I., Tacchini M., El Samrani A., Medawar S., Makhlouf H., Tannoury M. (2018). Phytochemical and pharmacological properties of essential oils from Cedrus species. Nat. Prod. Res..

[62] (1985). Spices and HERBS—Determination of Water Content—Entrainment Method. Spices and Aromatics—Determination of Water Content—Entrainment Method.

[63] (1972). Fruits, Vegetables and Derived Products—Mineralization of Organic Matter—Method by Incineration.

[64] (1999). Eurocode 9—Design of Aluminum Structures—Part 1-1: General Rules.

[65] Lowry O.H., Rosebrough N.J., Farr A.L., Randall R.J. (1951). Protein Measurement with the Folin Phenol Reagent. J. Biol. Chem..

[66] Senhaji S., Lamchouri F., Boulfia M., Lachkar N., Bouabid K., Toufik H. (2022). Mineral Composition, in Vitro Inhibitory Effects of α-Amylase, α-Glucosidase, β-Galactosidase Enzymes and Antibacterial Activity of Ajuga Iva Subsp. Pseudoiva (Dc.) Bric. Biointerface Res. Appl. Chem..

[67] Akrout A., Chemli R., Chreïf I., Hammami M. (2001). Analysis of the essential oil of *Artemisia campestris* L.. Flavour Fragr. J..

[68] Adams R.P. (2007). Identification of Essential Oil Components by Gas Chromatography/Mass Spectrometry.

[69] Kovats E.S. (1965). Gas chromatographic characterization of organic substances in the retention index system. Adv. Chromatogr..

[70] Balouiri M., Sadiki M., Ibnsouda S.K. (2016). Methods for in vitro evaluating antimicrobial activity: A review. J. Pharm. Anal..

[71] Liu M.H., Otsuka N., Noyori K., Shiota S., Ogawa W., Kuroda T., Hatano T., Tsuchiya T. (2009). Synergistic Effect of Kaempferol Glycosides Purified from *Laurus nobilis* and Fluoroquinolones on Methicillin-Resistant *Staphylococcus aureus*. Biol. Pharm. Bull..

