# Supplementary figures and images for: Phytochemistry and Biological Activities of Essential Oils from Six Aromatic Medicinal Plants with Cosmetic Properties
Source: Antibiotics (Basel). 2023 Apr 7;12(4):721. doi: 10.3390/antibiotics12040721 (PMC10135202; doi:10.3390/antibiotics12040721)

# Supplementary material

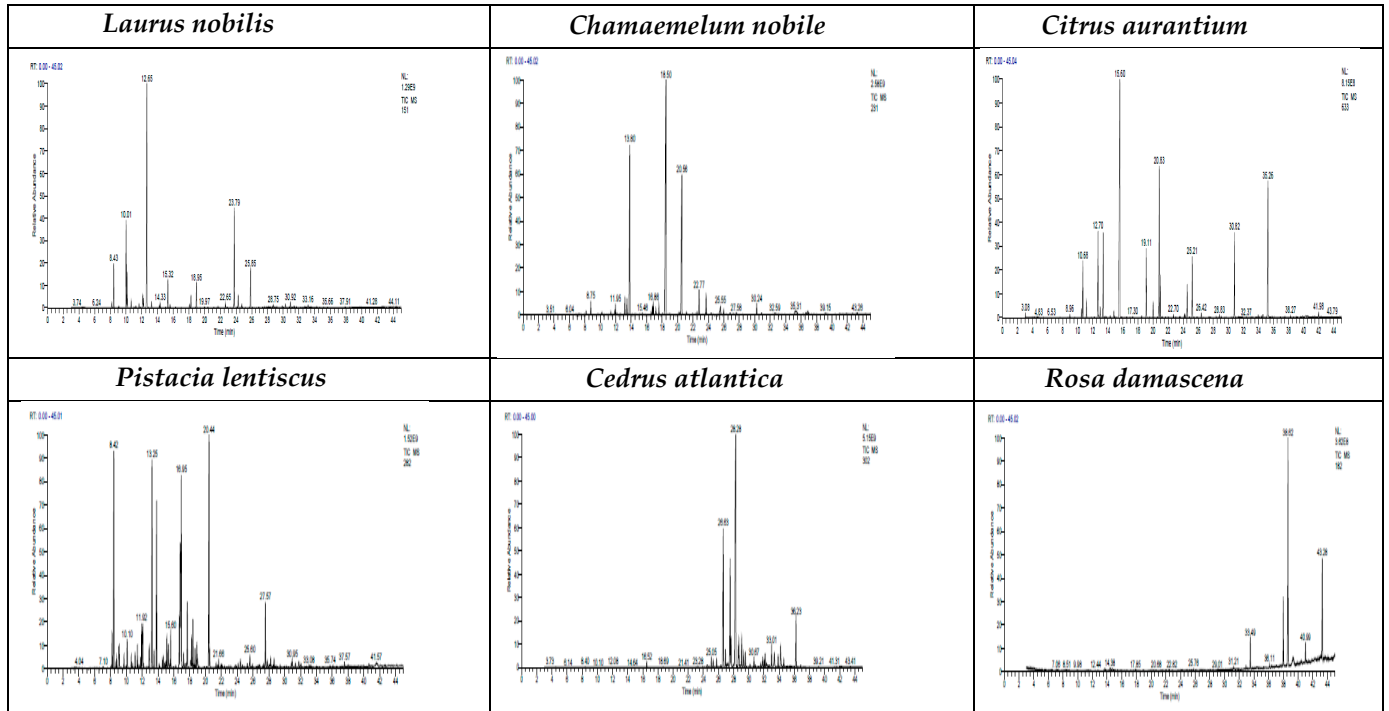

Figure S1: Representative GC–MS chromatogram of EOs of plants species.

Supplement: Supplementary file 1 [file antibiotics-12-00721-s001.zip › antibiotics-2216380-supplementary.pdf]
